# An ensemble framework for identifying essential proteins

**DOI:** 10.1186/s12859-016-1166-7

**Published:** 2016-08-25

**Authors:** Xue Zhang, Wangxin Xiao, Marcio Luis Acencio, Ney Lemke, Xujing Wang

**Affiliations:** 1 Systems Biology Core, NHLBI, NIH, 9000 Rockville Pike, Bethesda, MD 20892 USA; 2 Department of Computer Science, XiangNan University, Eastern Wangxian Park, Chenzhou, Hunan 423000 China; 3 Department of Physics and Biophysics, Institute of Biosciences of Botucatu, UNESP-São Paulo State University, CEP 18618-970, Botucatu, São Paulo 510 Brazil; 4 Department of Cancer Research and Molecular Medicine, Norwegian University of Science and Technology (NTNU), P.B. 8905, N-7491 Trondheim, Norway

**Keywords:** Essential protein, Protein-protein interaction networks, Centrality measure, Ensemble learning, Gene expression

## Abstract

**Background:**

Many centrality measures have been proposed to mine and characterize the correlations between network topological properties and protein essentiality. However, most of them show limited prediction accuracy, and the number of common predicted essential proteins by different methods is very small.

**Results:**

In this paper, an ensemble framework is proposed which integrates gene expression data and protein-protein interaction networks (PINs). It aims to improve the prediction accuracy of basic centrality measures. The idea behind this ensemble framework is that different protein-protein interactions (PPIs) may show different contributions to protein essentiality. Five standard centrality measures (degree centrality, betweenness centrality, closeness centrality, eigenvector centrality, and subgraph centrality) are integrated into the ensemble framework respectively. We evaluated the performance of the proposed ensemble framework using yeast PINs and gene expression data. The results show that it can considerably improve the prediction accuracy of the five centrality measures individually. It can also remarkably increase the number of common predicted essential proteins among those predicted by each centrality measure individually and enable each centrality measure to find more low-degree essential proteins.

**Conclusions:**

This paper demonstrates that it is valuable to differentiate the contributions of different PPIs for identifying essential proteins based on network topological characteristics. The proposed ensemble framework is a successful paradigm to this end.

**Electronic supplementary material:**

The online version of this article (doi:10.1186/s12859-016-1166-7) contains supplementary material, which is available to authorized users.

## Background

Genome-wide gene deletion studies show that a small fraction of genes in a genome are indispensable to the survival or reproduction of an organism [1, 2]. These genes are referred to as essential genes, and essential proteins are just the products of essential genes. The deletion of such essential proteins will result in lethality or infertility. Studies have shown that essential genes contribute to a diverse spectrum of diseases [3, 4]. Therefore, the identification of them is very important not only for understanding the minimal requirements for survival of an organism, but also for finding human disease genes [3, 4] and new drug targets [5, 6]. Several experimental essential proteins discovery methods have been developed, such as single gene knockouts [7], RNA interference [8] and conditional knockouts [9]. However, these experimental methods are very costly, time-consuming and laborious.

With the development of high-throughput experimental technologies, such as Y2H and mass spectrometry, large amounts of protein-protein interaction (PPI) data have been produced, which makes it possible to study proteins from network level. In order to overcome the experimental constraints, recently researchers have paid more attention to computational methods based on PPI network topological characteristics. These computational methods aim to mine and capture the correlations between network topological features and protein essentiality. It has been observed in the PINs of many species, such as *Saccharomyces cerevisiae*, *Caenorhabditis elegans*, and *Drosophila melanogaster* [10, 11], that proteins highly connected with other proteins are more likely to be essential than those selected by chance [12]. This phenomenon is referred to as the centrality-lethality rule [12], which demonstrates a high correlation between a node’s topological prominence in a PIN and its essentiality. Several researchers have begun to explain this rule in different hypotheses [13–16]. Although some controversies still exist about whether, why and how the highly connected proteins tend to be essential in PINs [13–16], most researchers have confirmed the correlation between topological centrality and protein essentiality [11, 17].

Besides the degree centrality (DC) [12], many other centrality measures, such as betweenness centrality (BC) [18], closeness centrality (CC) [19], eigenvector centrality (EC) [20], and subgraph centrality (SC) [21], have also been proposed to capture the correlations between network topological properties and protein essentiality. Betweenness centrality of a node is defined as the average fraction of the shortest paths that pass through the node. Joy et al. [18] have found that proteins with high betweenness are more likely to be essential. CC measures how quickly a node communicates with other nodes in the network. It is defined as the reciprocal of the average shortest distances from a node to all other nodes. EC is a measure of the influence of a node in a network. It is defined as the principal eigenvector of the adjacency matrix defining the network [20]. SC counts the total number of closed walks in which a protein participates in the PIN and gives more weights to closed walks of short lengths [21]. It has been confirmed that these topological properties correlate with the essentiality of proteins to some degree [12, 18, 22, 23]. Some recently proposed methods are also based on the topological properties of PINs, such as TP [24] and LAC [25], and they outperform the mentioned commonly used centrality measures. CytoNCA, a Cytoscape plugin, implemented some commonly used centrality measures as well as some newly proposed ones [26].

While these proposed centrality measures have demonstrated that network topological properties correlate with protein essentiality, low accuracy and low overlap exist when separately using these centrality measures to predict protein essentiality. It is expected that protein essentiality relates to multiple biological factors. Each of the proposed centrality measures often captures only one or a few topological properties which correlate with the protein essentiality. In addition, the currently available PINs for each species is incomplete (false negatives) and noisy (false positives), which further hinders the performance of these centrality measures.

In order to improve the prediction accuracy of essential protein discovery methods, biological and data quality information have been integrated with network topological properties, such as gene expression, cellular localization, biological process information, protein complex, and PPI confidence [22, 23, 27–33]. More specifically, due to the high number of false positives in PINs, the quantitative information of PPI confidence is believed to impact on essential protein discovery. As reported in [28], centrality measures can detect more essential proteins in weighted PINs (where edge weight is defined by the confidence of the corresponding PPI) than in their corresponding unweighted PINs (e.g., about 20 % improvement is obtained from CC and SC). However, although many methods have been proposed to evaluate the confidence of PPIs, as discussed by Li et al. [28], no more than 20 % improvement can be obtained for six centrality measures (DC, BC, EC, SC, CC and Information centrality) by considering the confidence of PPIs. The advances and challenges in identifying essential proteins using computational methods are reviewed in recent articles [34, 35].

According to the explanations for hub proteins’ essentiality either in the uniform essential PPI assumption [14] or in the essential complex assumption [15], different PPIs between a protein and its interacting neighbors are expected to have different contributions to the protein’s essentiality. The contribution of a PPI to its interacting proteins’ essentiality may relate to the confidence of the PPI, but is expected to be different from the confidence. For example, a PPI of high confidence is not necessarily having high contribution to the essentiality of its interacting proteins. Therefore, in terms of identifying essential proteins, how to evaluate the PPIs’ importance or contribution to their interacting proteins’ essentiality is non-trivial.

As we know, aside from technologically produced false positives, PINs provide in fact a set of putative interactions occurring between gene products. In the biological sense, these interactions are physically possible, but we don’t know if they occur inside the cell. This can be due to a number of possibilities, for instance, the protein pairs may not share the same cellular compartment, could not be expressed simultaneously, the interaction could occur but be biologically irrelevant, the interaction happen for short intervals. The integration of PINs with gene expression data is to provide a more reliable approximation to an in-vivo scenario. Recently several essential protein discovery methods by integrating PINs with gene expression data have been proposed, which outperform the centrality measures solely based on PINs. Most of these methods are based on two or more of the following assumptions: (1) A highly connected protein is more likely to be essential than a low connected one; (2) essential proteins tend to form densely connected clusters; (3) essential proteins in the same cluster have a higher chance to be co-expressed; (4) party hubs and date hubs have the similar probability to be essential while they have very different clustering property. PeC integrates edge clustering coefficient with gene co-expression correlation so as to capture both the co-clustering and the co-expression properties of a protein with its neighbors [22]. In CoEWC, a protein’s essentiality is determined by the number of the protein’s neighbors and the probability that the protein is co-expressed with its neighbors as well as its neighbors’ clustering properties [23]. Xiao et al. proposed a method to predict essential proteins based on active PPI network (APPIN) which is constructed based on static PPI network and dynamic gene expression data using both time-dependent and time-independent models, and applied several centrality measures in the APPIN to predict essential proteins [36]. The above mentioned integrated methods show that gene expression data can indeed be used to improve the accuracy of essential protein prediction from PINs, and they also shed light on how to integrate it with PINs. Generally speaking, different assumptions can lead to different integration strategies, which in turn can be of different prediction accuracy.

In this paper, we propose an ensemble framework aiming to increase the prediction accuracy of basic centrality measures. It integrates biological information (gene expression) with PINs. In our framework, a series of PINs are generated based on the original one using a gene expression correlation-guided bagging strategy. Five centrality measures (DC, BC, EC, SC, and CC) are applied in the PINs and a set of scores for each protein are obtained according to each centrality measure. A final score is computed for each protein by using a weighted voting strategy, which reflects the probability of a protein being essential. Differing from other proposed methods, a protein’s essentiality in our ensemble framework is determined not only based on its connectivity in the original PIN [12, 18], but also by its connectivity in a series of generated PINs. The performance of the ensemble framework was tested on the protein interaction networks of *Saccharomyces cerevisiae*, a well-studied species by knockout experiments.

This paper is organized as follows. We first show the materials used to test our framework, including the protein-protein interaction data, gene expression data, and the information of gene essentiality. Then the proposed framework is depicted in detail. The experimental results are presented and discussed in the following section. Finally we present our conclusion and future directions.

## Methods

In this paper, a PIN is represented by an undirected graph *G*(*V*, *E*), where a node *v*∈*V* represents a protein and an edge *e*(*u,v*)∈*E* denotes an interaction between two proteins *u* and *v*. In the context of PINs, protein centrality is used to characterize the importance or contribution of an individual protein to the global structure or configuration of the PIN [37]. In the PIN, each protein is assigned a score indicating its relative possibility to be essential according to each centrality measure. Top ranked proteins are taken as candidates of essential proteins.

### Test data

To evaluate the performance of the ensemble framework, the PPI and gene expression data of *Saccharomyces cerevisiae* were used. This model organism has been characterized by knockout experiments and widely used in the evaluation of essential protein discovery methods. The test data used in this paper are as follows.

The PPI data were downloaded from the DIP [38] and BioGRID (release 3.2.99) databases [39]. From DIP, 5093 proteins and 24,743 interactions are obtained after removing self-interactions and repeated interactions. From BioGRID, there are 76,610 unique physical interactions among 6074 proteins in total after genetic interactions, self-interactions and repeated interactions were filtered. 4731 proteins are common among the two PPI data sets. For convenience, we refer to the PIN from DIP database as PIN24K, and the PIN from BioGRID database as PIN76K in the following.

The Saccharomyces genome deletion consortium reports a total of 1156 essential open reading frames (ORFs) [1], among which 1122 are unique. Among the 1122 corresponding essential proteins, there are 1024 involved in the PIN24K and 1045 involved in the PIN76K. We refer to them as standard-1122 for convenience. Other essential gene collections include DEG [40] and SGD [41]. In DEG, there is a collection of 1110 yeast essential genes, but only 1037 and 1100 are involved in the PIN24K and PIN76K, respectively. In SGD, there is a collection of 1279 yeast essential proteins, where 1159 and 1201 essential proteins are included in the PIN24K and PIN76K, respectively. In the following analysis, standard-1122 is used as the reference for protein’ essentiality, unless otherwise stated. The other two essential protein collections are used as further references.

The gene expression data of *Saccharomyces cerevisiae* were retrieved from [42], which have been used for the task of identifying essential proteins by other authors [22, 23, 36]. It contains 6777 genes and 36 samples in total. There are 4985 and 5433 proteins included in this gene expression data from PIN24K and PIN76K, respectively. For proteins which have no corresponding gene expression data, we simply set them with zero values. Although many collections of gene expression data for *Saccharomyces cerevisiae* exist, most of them either have small sample size (i.e., few conditions for capturing gene expression levels) or are devoted to specific special treatments. The collection of gene expression data from [42] spans three cell cycles and has a large coverage of yeast genes. Comparatively speaking, we think this collection is more suitable for the task of identifying essential proteins.

### The proposed ensemble framework

In machine learning, ensemble methods use multiple models to obtain better predictive performance than could be obtained from any of the constituent models alone [43]. Bootstrap aggregating, often abbreviated as bagging, is a popular ensemble technique which involves having each model in the ensemble vote with equal weight. In order to promote model variance, bagging trains each model in the ensemble using a randomly drawn subset of the training set. In bagging, models can be trained in parallel. Boosting is another ensemble technique which involves incrementally building an ensemble by training each new model to emphasize the training instances that previous models misclassified. In boosting, models are trained in sequence.

If we take protein-protein interactions (PPIs) in a PIN as the training information, false positive PPIs as noise introduced in the training information, and each centrality measure as the base learning model, then we can construct an ensemble framework for each centrality measure with the expectation of an improved performance just as it does in other application domains. The ensemble framework in this study aims to lessen the effect of noise contained in the PPIs as well as to emphasize the effect of PPIs which are considered important for their interacting proteins’ essentiality. To this end, we construct the ensemble framework by using a correlation-guided sampling method which combines the thoughts of both bagging and boosting.

The ensemble framework consists of the following four steps. (1) ***Data partition***: from the original PPI data, a series of, say *m*, PINs, are constructed according to certain criteria (see below). This step is similar to that used in bagging to generate training data for each of the ensemble models. (2) ***Grading***: compute a score for each protein on each of the *m* generated PINs using a centrality measure. Then we can get *m* scores for each protein by using each centrality measure. (3) ***Integrating***: integrate the *m* scores for each protein into one final score according to some strategy (see below). (4) ***Ranking***: rank the proteins according to their final scores. For the top *n* ranked proteins, more true essential proteins contained, more effective the ranking method is.

### Data partition

Data partition is one of the most important steps in the proposed ensemble framework. In this paper, we use a correlation-guided data partition strategy. The basic ideas behind this data partition strategy include: (a) essential proteins are more likely to be co-expressed with some of their neighbors; (b) PPIs between two highly co-expressed proteins are more important to the proteins’ essentiality. Pearson correlation coefficient (PCC) is used to evaluate how strong two interacting proteins in a PIN are co-expressed.

**Fig. 1:**
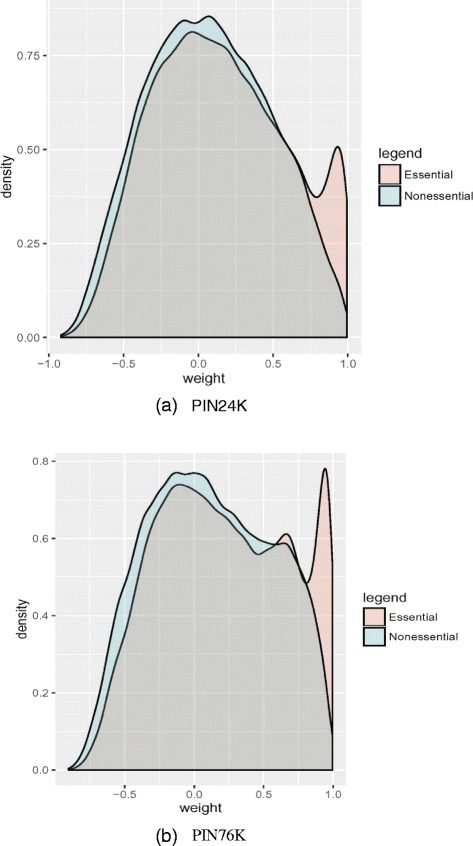
The distributions of co-expression weights for essential and nonessential proteins. The co-expression weight for each protein pair was calculated as the Pearson correlation coefficient

We evaluated the data to see if essential proteins tend to display more co-expressed partners in the PINs (assumption a). Figure 1 gives the distributions of co-expression weights (Pearson correlation coefficients) for both essential and nonessential proteins. Evidently the weight distributions are significantly different between essential and nonessential proteins (KS test p-value < 2.2e-16 on both PIN24K and PIN76K). Essential proteins indeed tend to be co-expressed with their partners compared with nonessential proteins. We further examined the node strength distributions of essential and nonessential proteins (Additional file 1: Figure S1) and noticed a significant difference between the two distributions (KS test p-value < 2.2e-16).

According to [14], centrality-lethality rule can be explained based on essential PPIs. The authors stated that some PPIs are more important than others which would be particularly meaningful if there are PPIs that are essential to the survival or reproduction of an organism. An essential interaction between two proteins makes both proteins essential because the removal of either protein causes lethality or infertility due to the disruption of the interaction. For example, yeast proteins SPT16 and POB3 are both essential and they form heterodimers that function in DNA replication. Genetic studies showed that their interaction is critical for this function [44]. Two proteins forming an essential PPI both must be essential, but interactions between essential proteins (IBEPs) may or may not be essential because the essentiality of a protein can be due to factors other than essential PPIs. The authors found that about 3 % yeast PPIs are essential PPIs based on random rewiring method [14]. Additional file 1: Figure S2 gives the distributions of co-expression weights for IBEPs and Non-IBEPs, which tell us that these two types of PPIs have significantly different co-expression weights distributions (KS test p-value < 2.2e-16). We found that the IBEPs, whose co-expression weights larger than 0.75, account for about 3.9 and 3 % yeast PPIs based on BioGRID and DIP respectively. This proportion consists with that of essential PPIs reported in [14]. We propose that IBEPs with high co-expression weights may be more likely to be essential PPIs, although there’s no easy way to validate this assumption. Based on these circumstantial evidences, we proposed our second assumption that PPIs between two highly co-expressed proteins are more important to the proteins’ essentiality.

The *data partition strategy* works as follows. First, it computes the PCC value for each pair of directly interacting proteins in the original PIN. Second, a series of thresholds are determined according to the distribution of PCC values of all the PPIs in the original PIN where maxPCC (minPCC) is the maximal (minimal) PCC value among all PPIs’ in the original PIN. There are two ways to generate the thresholds. One is the absolute thresholding strategy, in which we have each threshold *thr*_*i*_∈[0, maxPCC), *i* = 1,2,…,*m*. The other is so-called uniform thresholding strategy, in which we have each threshold *thr*_*i*_∈(minPCC, maxPCC), *i* = 1,2,…,*m*. Third, according to each thresholding strategy, *m* PINs are generated from the original PIN. In the absolute thresholding strategy, for each threshold *thr*_*i*_, the PPIs, whose absolute PCC values are below the threshold, are deleted from the original PIN, and a new PIN, say PIN_*i*_, in which the absolute PCC values between all directly interacting protein pairs are larger than the threshold *thr*_*i*_, is generated. Then *m* PINs are generated from the original PPI data. Similarly, *m* PINs can be generated by using the uniform thresholding strategy. For each threshold *thr*_*i*_, PIN_*i*_ is generated by deleting all PPIs whose PCC values are less than *thr*_*i*_ from the original PIN.

The data partition process, which is guided by the correlation, is different from the random sampling strategy for training data generation in bagging. Instead, it is similar with that of boosting since both of them select training data under the guidance of some knowledge. On the other hand, the PINs are all generated before applying learners (centrality measure, in this case), which is similar with that of bagging, and so the following training and predicting (compute the scores according to certain centrality measure, in this case) processes can be executed in parallel. Therefore, the data partition strategy integrates the advantages of both bagging and boosting.

### Grading

In this paper, five commonly used centrality measures are employed, and their performance are compared with and without the ensemble framework. These five centrality measures are degree centrality (DC) [12], betweenness centrality (BC) [18], closeness centrality (CC) [19], eigenvector centrality (EC) [20], and subgraph centrality (SC) [21].

In the grading stage, one set of *m* scores are calculated for each protein according to each centrality measure on each PIN (*m* generated PINs in data partition stage). For example, if we take DC as the base centrality measure, then for each protein, there are *m* DC scores for it calculated from the *m* PINs respectively. Note that, according to the data partition strategy, the magnitudes of the *m* scores for each protein generally decreases with the increase of thresholds for generating the PINs, that is, the score of a protein calculated on PIN_*i*_ is generally larger than that computed on PIN_*i*+1_ for the same protein according to the same centrality measure. The scores for all proteins computed from the same PIN using the same base centrality measure are normalized by dividing their maximal value. The normalization aims to make scores calculated from different PINs comparable and to make it convenient for the integrating stage.

### Integrating

In ensemble learning, multiple predictions by ensemble models are often combined in some way (typically by weighted or unweighted voting) to classify new examples. In this paper, we use a weighted voting strategy to combine the scores computed on the *m* PINs for each centrality measure.

According to the basic ideas behind the data partition strategy, larger weights should be given to the scores computed from the PINs generated with larger thresholds. Formally, let *w*_*i*_ be the weight assigned to PIN_*i*_, then *w*_*i*_ ≤ *w*_*i*+1_, *i* = 1,…,*m*-1. Let *s*(*j*,*i*) be the score calculated on PIN_*i*_ for protein *j* according to a centrality measure, then the final score, *fs*(*j*), for protein *j* in the ensemble framework is calculated by

1 $$ fs(j)={\displaystyle {\sum}_{i=1}^ms\left(j,i\right)\times {w}_i}. $$

The ensemble framework aims not only to emphasize PPIs which have more contributions to protein essentiality, but also to reserve useful information of the original PIN to the greatest extent. With the increase of thresholds, more and more PPIs are deleted from the original PIN, and some useful information may also be lost. Therefore, we would set the weight of the original PIN to 1 to alleviate such information lost.

### Ranking

In the ensemble framework, proteins are ranked according to their final scores in descending order. Top *n* ranked proteins are taken as the candidates of essential proteins, out of which the number of true essential proteins are determined according to the list of known essential proteins. Therefore, the more essential proteins that are contained in the top *n* candidates, the better the prediction or ranking method is.

## Results and discussion

For convenience, the ensemble framework using DC (BC, EC, SC and CC) as the base model is referred to as EnDC (EnBC, EnEC, EnSC and EnCC, respectively). EnDC-a denotes the ensemble method using absolute thresholding strategy and EnDC-u denotes the ensemble method using uniform thresholding strategy. The same goes for other four ensemble methods: EnBC, EnCC, EnEC and EnSC.

In the following experiments, we set *thr*_*i*_ = {0,0.1,0.2,…,0.8,0.9,0.91,0.92,…,0.95} for the absolute thresholding strategy, and *thr*_*i*_ = {-0.7,-0.6,…,0,0.1,0.2,…,0.8,0.9,0.91,0.92} for the uniform thresholding strategy. Therefore, in the ensemble framework, there are *m* = 15 PINs generated using absolute thresholding strategy while *m* = 19 PINs using uniform thresholding strategy.

### Performance of the ensemble framework

#### Two thresholding strategies

We first conducted experiments to evaluate the performance of these two thresholding strategies. The weight vector w = {1, 2, 3, 5, 8, 13, 21, 34, 55, 89, 144, 233, 377, 610, 987} for ensemble methods with absolute thresholding strategy, and w = {1, 1, 1, 1, 1, 1, 1, 1, 2, 3, 5, 8, 13, 21, 34, 55, 89, 144, 233} for ensemble methods with uniform thresholding strategy.

**Fig. 2:**
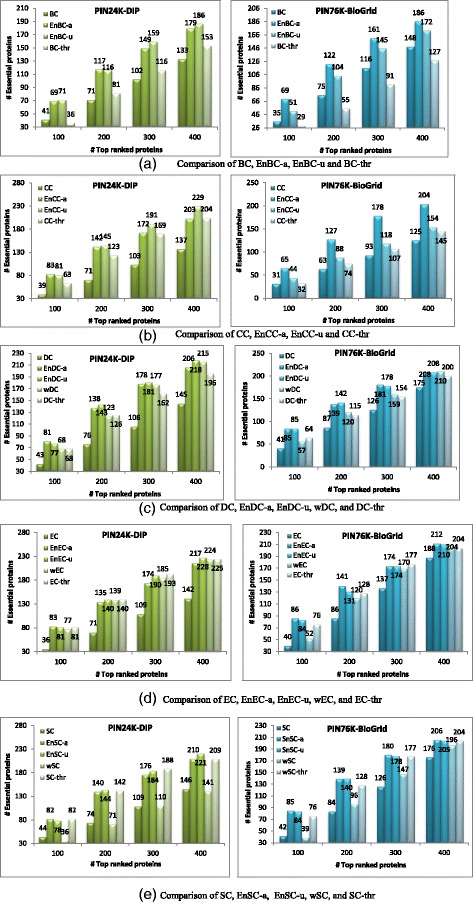
Performance comparison of two thresholding strategies of the ensemble framework on two yeast PINs. Five centrality measures (BC, CC, DC, EC and SC) are used as the base models respectively. We also compared our ensemble methods with: 1) using a single PCC-thresholded PIN with thr = 0.75, and 2) using a PCC-weighted version of PIN. Proteins are ranked according to their final scores calculated by each ensemble method (i.e., EnBC, EnCC, EnDC, EnEC and EnSC) or by each centrality measure (BC, CC, DC, EC and SC) or by the other compared methods. For each ensemble method on each case, top *n* ranked proteins are selected as the candidates of essential proteins, out of which the number of true essential proteins are determined. The same procedure is applied to five centrality measures and the other compared methods

Figure 2 gives the performance comparison of the ensemble framework with two thresholding strategies on two yeast PINs, PIN24K (DIP) and PIN76K (BioGRID). Five centrality measures (BC, CC, DC, EC and SC) are used as the base models to test whether or how much they can benefit from the proposed ensemble framework. From Fig. 2 we can see that the two thresholding strategies perform similarly on PIN24K, while EnBC-a and EnCC-a perform better than their corresponding ensemble methods, EnBC-u and EnCC-u, on PIN76K. In top *n* ranked proteins, the ensemble methods, using either thresholding strategy, considerably outperform their corresponding centrality measures. This validates the usefulness of the ensemble framework for identifying essential proteins from PINs. For example, in top 100 ranked proteins, more than 100 % improvement is obtained by four ensemble methods (EnCC-a, EnDC-a, EnEC-a, and EnSC-a) on PIN76K and by two ensemble methods (EnCC-a and EnEC-a) on PIN24K, as compared with their corresponding centrality measures. EnDC-a and EnSC-a achieve an 85 % improvement on PIN24K as compared with DC and SC, respectively. EnBC-a compared with BC achieves the least improvement, but there are still 68 % improvement on PIN24K and 97 % improvement on PIN76K, respectively.

We also compared our ensemble methods with two PCC based methods: applying centrality measures in PCC-threshold PIN, and in PCC-weighted PIN. For PCC-threshold method, we set threshold to 0.75, which is based on the distribution of co-expression weights distribution and IBEP weights distribution (Fig. 1 and Additional file 1: Figure S2) because at this point essential proteins and IBEPs start having different co-expression weight distributions compared with nonessential proteins and Non-IBEPs. PCC-threshold PIN was generated by removing PPIs whose absolute PCC values are less than the threshold from the original PIN. PCC-weighted PIN was generated by adding weight to each PPI in the original PIN using its corresponding PCC value. Then five centrality measures were applied in the two PINs respectively. We use M-thr to denote PCC-threshold method and wM to denote PCC-weighted method, where M is one of the five centrality measures. The performance of these two methods is also included in Fig. 2. Note that BC and CC can only be applied in graphs with positive edge weights, so they are not suitable for PCC-weighted method. Figure 2 tells us that ensemble methods EnBC, EnCC, and EnDC outperform the PCC-threshold methods BC-thr, CC-thr, and DC-thr, while they perform similarly when using EC and SC as base learners. The ensemble methods EnDC, EnEC, and EnSC outperform PCC-weighted methods wDC, wEC, and wSC especially with smaller *n* in top *n* candidates.

**Fig. 3:**
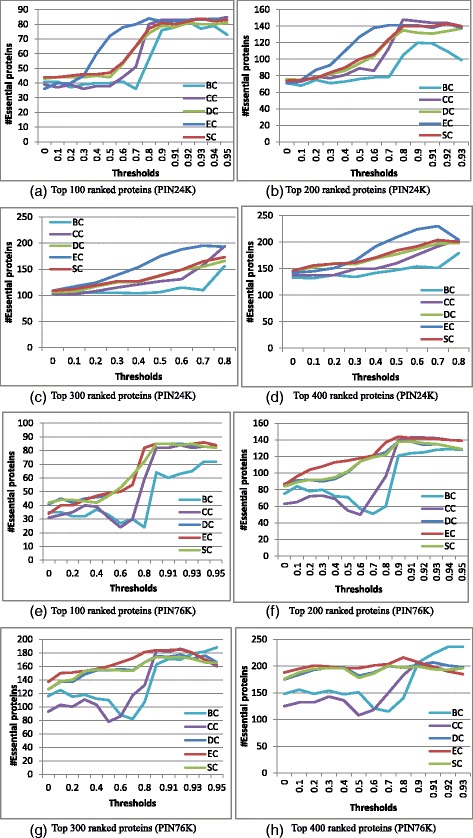
Performance comparison of five centrality measures (BC, CC, DC, EC, and SC) on two yeast PINs (PIN24K and PIN76K) using absolute thresholding strategy. For each yeast PIN (PIN24K or PIN76K), 15 PINs are generated by using the absolute thresholding strategy. Proteins are ranked according to their values calculated by each centrality measure on each PIN. For each centrality measure on each PIN, top *n* proteins are selected as candidates for essential proteins, out of which the number of true essential proteins are determined. The PIN with threshold 0 is the original PIN (PIN24K for (**a**)-(**d**), and PIN76K for (**e**) ~ (**h**)). X-axis represents the thresholds and y-axis the number of true essential proteins in top *n* ranked proteins. Proteins contained in standard-1122 are considered as essential proteins

Figure 3 and Additional file 1: Figures S3-S5 presents the performance comparison of five centrality measures (BC, CC, DC, EC, and SC) with different thresholds by using two thresholding strategies on two yeast PINs (PIN24K and PIN76K), as well as the relationship between the number of nonzero-degree nodes and the thresholds for generating the corresponding PINs when applying two thresholding strategies. In Fig. 3 and Additional file 1: Figure S3, we only show the performance of five centrality measures with thresholds that the number of nonzero-degree nodes in their corresponding PINs is larger than *n* when top *n* ranked proteins are considered as essential protein candidates.

As shown in Fig. 3 and Additional file 1: Figure S3, the performance of five centrality measures varies slightly at small threshold values in both thresholding strategies. Then their performance increases rapidly when the thresholds are larger than 0.7 and almost reaches their best performance when the thresholds are larger than 0.8. It can be seen that the number of true essential proteins in the top 100 ranked proteins on the PINs generated with thresholds larger than 0.8 becomes almost twice of that of the original PIN for each centrality measure. In top *n* ranked proteins for absolute thresholding strategy, five centrality measures benefit more with smaller *n*, and such benefits decrease with the increase of *n*. For uniform thresholding strategy, the performance of the five centrality measures has almost no change with thresholds smaller than zero, and then the performance increases with the increase of thresholds similarly to that in the absolute thresholding strategy. Therefore we set the weights to 1 for negative thresholds in the uniform thresholding strategy and set the weights for other thresholds the same as in the absolute thresholding strategy in the ensemble framework (see Fig. 2). The performance dependence on thresholds further confirm our basic ideas behind the ensemble framework: (1) essential proteins tend to be co-expressed with some of its neighbors, and (2) PPIs between two highly co-expressed proteins are more important to the proteins’ essentiality. Therefore, the performance of five centrality measures is improved by deleting trivial PPIs whose interacting proteins’ expression is not correlated.

Furthermore, it can be seen that the five centrality measures on the generated PINs performs similarly or even outperforms their ensemble counterparts at some thresholds (see Fig. 3, Additional file 1: Figure S3, and Fig. 2). One may question why we should use the ensemble framework. As shown in Additional file 1: Figure S4 and Figure S5, the number of nonzero-degree proteins in the generated PINs decreases with the increase of the thresholds for generating the PINs. For example, only 290 and 588 nonzero-degree proteins are left in the PINs generated from PIN24K and PIN76K respectively when the threshold equals to 0.9 in absolute thresholding strategy. Only these nonzero-degree proteins would be given nonzero scores by five centrality measures so that they can be ranked according to their scores. The other proteins will be ranked randomly since they all have zero scores. Therefore, the thresholding method might be useful when we only want to select a small number of proteins so as to study their essentiality. However, the ensemble framework aims to discover most of the essential proteins by making the best use of useful information contained in the original PIN while at the same time highlighting some PPIs which are more important for proteins’ essentiality. The other disadvantage of PCC-threshold method is that we usually don’t know which threshold is better for a new organism. Although we can select the threshold based on experiences from other well studied organisms, there should be difference between organisms. Furthermore, from Fig. 2, we can see that PCC-threshold methods don’t always have similar performance with ensemble methods for different centrality measures.

In the following we take only the ensemble framework with the absolute thresholding strategy into consideration. A similar analysis can be obtained for the uniform thresholding strategy. One aspect of the absolute thresholding strategy is the selection of thresholds. From a biological perspective, it might not make sense to differentiate PCC values between 0.91 and 0.92. In Additional file 1: Figure S4 we can also see that the number of nonzero-degree proteins changes very slowly with the increase of thresholds from 0.9 to 0.95 on two yeast PINs (PIN24K and PIN76K). However, please note that this ensemble framework aims to emphasize the effect of some PPIs which are expected to be important for their interacting proteins’ essentiality and that PPIs between two highly co-expressed proteins are considered to be more important to the proteins’ essentiality. The results shown in Fig. 3 and Additional file 1: Figure S3 support our view. The PINs generated with thresholds larger than 0.9 should mainly contain the PPIs which connect highly co-expressed proteins and are more informative for identifying essential proteins according to the basic ideas of the ensemble framework. Therefore, five centrality measures on the PIN generated with threshold 0.9 considerably outperforms their corresponding counterparts on the original PIN.

There are multiple methods to emphasize the information contained in the PINs generated with larger thresholds. One is the dense sampling method which samples more PINs with larger thresholds as shown in the ensemble framework with the absolute thresholding strategy, and the other method is dominant weighting which gives dominant weights to such PINs. Additional file 1: Figure S6 presents the experimental results of these two methods. In Additional file 1: Figure S6, we use M (0 ~ 0.9) to test the dominant weighting in which the PIN generated with the largest threshold (0.9, in this case) referred to as the dominant PIN, and we use M (0 ~ 0.95) to test dense sampling. M is one of the five ensemble methods: EnBC-a, EnCC-a, EnDC-a, EnEC-a, and EnSC-a. Of course more dominant PINs can be chosen, but for simplicity we only choose one dominant PIN here. From Additional file 1: Figure S6 we can see that ensemble methods using the dominant way achieve better performance by giving larger weight to the dominant PIN. On PIN24K, two ensemble methods (EnCC-a and EnEC-a) can achieve almost the same performance for both methods, while the other three ensemble methods perform slightly better using dense sampling. On PIN76K, three ensemble methods (EnDC-a, EnEC-a and EnSC-a) perform similarly for both methods, while other two ensemble methods (EnBC-a and EnCC-a) perform better using dense sampling. In general, dense sampling is better than the dominant weighting, so in the following analysis we only consider the dense sampling approach, that is, the absolute thresholding strategy used in the ensemble framework.

### Voting weights

The proposed ensemble framework takes a weighted voting strategy to integrate the scores computed on different PINs. Based on the basic ideas behind the ensemble framework, the weighted voting strategy should favor the scores computed on the PINs generated with larger thresholds. That is, it favors the PPIs whose interacting proteins are highly co-expressed. This is based on the assumption that essential proteins tend to be highly co-expressed with some of their co-clustered neighbors. This assumption can be observed in the two yeast PINs (Fig. 1 and Additional file 1: Figure S2).

**Fig. 4:**
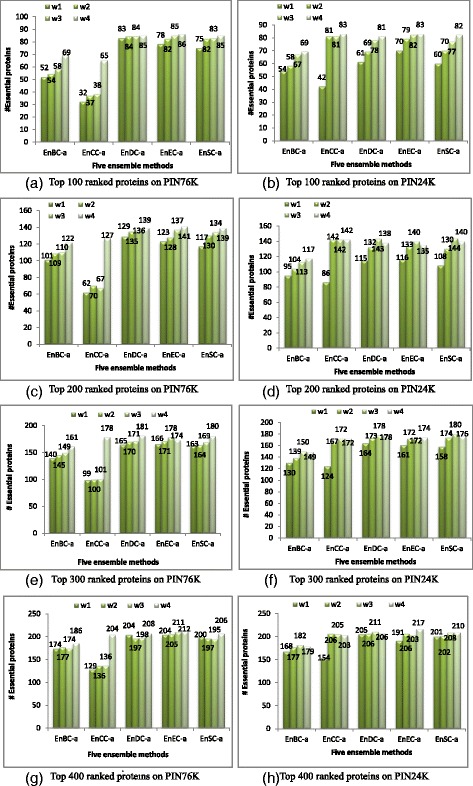
Comparison of the number of essential proteins detected by each ensemble method using absolute thresholding strategy with different weights on two yeast PINs

Figure 4 presents the performance comparison of five ensemble methods using absolute thresholding strategy with different types of voting weights. In Fig. 4, four types of voting weights are considered. w1 = {1, 1, 1, 1, 1, 1, 1, 1, 1, 1, 1, 1, 1, 1, 1}, delegates the unweighting strategy which doesn’t discriminate the contributions from different generated PINs, and is used as a base line. w2 = { 1, 2, 3, 4, 5, 6, 7, 8, 9, 10, 11, 12, 13, 14, 15}, is called gradual advance weighting. w3 = {1, 1, 1, 1, 1, 1, 1, 1, 8, 9, 10, 11, 12, 13, 15}, is called prior guided advance weighting. w4 = {1, 2, 3, 5, 8, 13, 21, 34, 55, 89, 144, 233, 377, 610, 987}, is called dominant weighting in which the weight for each generated PIN is the sum of its two preceding PINs’ weights. Gradual advance weighting (w2) and dominant weighting (w4) are designed based on the assumption that PPIs whose interacting proteins are highly co-expressed may be more important in identifying essential proteins. w3 is designed by the knowledge that all five centrality measures perform better on PINs generated with thresholds larger than 0.7 (see Fig. 3), so the first eight PINs (one original PIN and seven generated PINs with thresholds from 0.1 to 0.7) are given weights 1, and the weights increase gradually for the subsequent PINs generated with the thresholds lager than 0.7. Therefore gradual advance weighting and dominant weighting are feasible for the ensemble framework since they don’t require the information of performance distribution on the generated PINs.

In Fig. 4 we can see that dominant weighting is better than other three types of voting weights for all five ensemble methods. In addition, three ensemble methods (EnDC-a, EnEC-a and EnSC-a) are quite robust in selecting voting weights. EnCC-a and EnBC-a benefit more from the dominant weighting strategy, especially for EnCC-a on PIN76K. BC and CC are two global centrality measures, while DC, EC, and SC are local centrality measures. This tells us that global centrality measures are more sensitive to noisy and trivial PPIs (whose proteins are not co-expressed) for predicting protein essentiality than those local centrality measures.

Additional file 1: Figure S7 presents the performance comparison of five ensemble methods using uniform thresholding strategy with different types of voting weights. Similarly, four types of voting weights are adopted to test the performance of the ensemble framework with the uniform thresholding strategy. As shown in Additional file 1: Figure S7, prior guided dominant weighting (w4) is better than the other three types of voting weights for five ensemble methods. Prior guided advance weighting is in second place. By comparison of Fig. 4 and Additional file 1: Figure S7, we can further conclude that the absolute thresholding strategy is better than the uniform thresholding strategy. In the following analysis, we only consider the ensemble framework using the absolute thresholding strategy and dominant voting weights.

### Common predicted essential proteins

Although many centrality measures have been reported to have some correlation with protein essentiality, most of them have low accuracy in identifying essential proteins when used alone (Fig. 2). There are multiple reasons for the lack of accuracy, the incomplete and noisy PIN on which centrality measures depends, and the fact that protein essentiality is expected to relate to many biological features additional to one or few topological properties of the PIN. It is therefore not surprising that centrality measures have limited predictive ability for protein essentiality. Thus how to integrate useful topological information captured by different centrality measures, as well as different biological data sources is very important for identifying essential proteins. Furthermore, our experimental results on two yeast PINs show that the number of common predicted proteins (overlap) by five centrality measures is very small (see Table 1), in addition to their low accuracy.

**Table 1:** The number of common predicted proteins (overlap) among the top 100 proteins ranked by different centrality measures

Datasets	Centrality measures	BC		EC		SC		CC		#overlaps among five centrality measures	
		#overlap	#EP	#overlap	#EP	#overlap	#EP	#overlap	#EP	#overlap	#EP
PIN76K	DC	66	22	57	26	92	38	51	20	29	7
	BC			36	12	63	21	49	16		
	EC					65	30	41	12		
	SC							51	19		
PIN24K	DC	84	36	57	29	97	43	71	31	43	21
	BC			47	24	82	36	64	28		
	EC					56	29	67	29		
	SC							70	31		

Proteins are ranked according to the scores calculated by each centrality measure. Then top 100 ranked proteins for each centrality measure are selected as the candidates of essential proteins, out of which the number of true essential proteins are determined. #overlap represents the number of common predicted proteins among the top 100 ranked proteins by different centrality measures and #EP the number of true essential proteins among the overlaps

Table 1 gives the number of overlap among the top 100 proteins ranked by any two different centrality measures and the number of essential proteins among the overlap, as well as the number of overlap by all the five centrality measures and the corresponding number of essential proteins. As shown in Table 1, there is a larger overlap rate between DC and SC on both two yeast PINs (92/100 on PIN76K and 97/100 on PIN24K), which indicates the positive correlation between DC and SC. But the number of true essential proteins is small (38/92 on PIN76K and 43/97 on PIN24K) because of the low accuracy of DC and SC for predicting essential proteins. The overlap rate between BC and EC is the lowest (36/100 on PIN76K and 47/100 on PIN24K), so is the overlap rate for true essential proteins (12/36 on PIN76K and 24/47 on PIN24K). In general, true essential proteins only account for a small proportion of the overlap (24 % ~ 46 % on PIN76K, and 42 % ~ 51 % on PIN24K). The overlap rates are even lower among the five centrality measures on two yeast PINs (29/100 on PIN76K and 43/100 on PIN24K), let alone the number of essential proteins in the overlap (7 on PIN76K and 21 on PIN24K).

Table 2 gives the number of overlaps among the top 100 proteins ranked by different ensemble methods and the number of essential proteins among the overlaps. EnDC-a and EnSC-a have the largest overlap rate on two yeast PINs (99/100 on PIN76K and 98/100 on PIN24K), which is similar to their corresponding centrality measures, DC and SC. Out of the overlap between EnDC-a and EnSC-a, the overlap rate for essential proteins is also high (83/99 on PIN76K and 80/98 on PIN24K). EnDC-a and EnCC-a have the lowest overlap rate on PIN76K (40/100), while EnBC-a and EnEC-a have the lowest overlap rate on PIN24K (60/100). In general, among the overlap, true essential proteins account for a proportion about 67 % ~ 92 % on PIN76K and 78 % ~ 83 % on PIN24K, which are much higher compared with those of centrality measures. Although a slight decrease in the overlap rate exists for a few pairs of ensemble methods compared with those of their corresponding pairs of centrality measures, the overlap rate of true essential proteins increases. For example, the overlap between EnDC-a and EnBC-a decreases to 60 from 66, the overlap between DC and BC, but the overlap of essential proteins between EnDC-a and EnBC-a increases to 51 from 22. From Tables 1 and 2 we can see that the overlaps of top ranked proteins between ensemble methods contain more essential proteins than those between the centrality measures.

**Table 2:** The number of common predicted proteins (overlap) among the top 100 proteins ranked by different ensemble methods

Datasets	Ensemble methods	EnBC-a		EnEC-a		EnSC-a		EnCC-a		#overlaps among five ensemble methods	
		#overlap	#EP	#overlap	#EP	#overlap	#EP	#overlap	#EP	#overlap	#EP
PIN76K	EnDC-a	60	51	92	79	99	83	40	37	28	25
	EnBC-a			55	47	59	50	59	40		
	EnEC-a					93	80	41	37		
	EnSC-a							41	38		
PIN24K	EnDC-a	74	58	84	69	98	80	82	67	53	42
	EnBC-a			60	48	72	57	71	56		
	EnEC-a					86	71	72	60		
	EnSC-a							80	66		

Proteins are ranked according to the scores calculated by each ensemble method. The top 100 ranked proteins for each ensemble method are selected as the candidates for essential proteins, out of which the number of true essential proteins are determined. #overlap represents the number of common predicted proteins among the top 100 ranked proteins by different ensemble methods and #EP the number of true essential proteins among the overlaps

Additional file 1: Tables S1 and S2 give the number of overlap among the top 100 proteins ranked by two different PCC-weighted methods or by two different PCC-threshold methods. For PCC-weighted methods, among the overlap, true essential proteins account for a proportion about 42 % ~ 56 % on PIN76K and 56 % ~ 75 % on PIN24K. The overlap is lower on PIN24K than that on PIN76K. For PCC-threshold methods, true essential proteins account for a proportion about 36 % ~ 76 % on PIN76K and 50 % ~ 88 % on PIN24K. By comparing with Tables 1 and 2, we can see that ensemble methods have higher rates of true essential proteins among the overlaps.

We further analyzed how correlated the five centrality measures and the five ensemble methods are by using their top 100 ranked proteins. For each pairwise comparison, we use the union of their top 100 ranked proteins to calculate the Pearson correlation coefficient based on their corresponding scores. Additional file 1: Table S3 and S4 give the correlations between centrality measures and between ensemble methods. On PIN76K, EnBC-a and EnCC-a show almost no correlation with other ensemble methods while their corresponding centrality measure pairs have strong correlations, and three ensemble methods (EnDC-a, EnEC-a, and EnSC-a) show similar correlations compared with their corresponding centrality measures. On PIN24K, four ensemble pairs (EnBC-a and EnEC-a, EnBC-a and EnSC-a, EnDC-a and EnEC-a, EnDC-a and EnSC-a) show larger correlations compared with their corresponding centrality measures pairs. In general, the ensemble framework doesn’t significantly increase pairwise correlations, and it even decreases the pairwise correlations (for example, on PIN76K). We also can see that the correlation is not necessarily proportional to the overlap between two methods. For example, on PIN76K, there are 57 overlap between DC and EC, 92 overlap between EnDC-a and EnEC-a, but the correlations are similar or even a little decrease from DC and EC pair to the ensemble pair. Three local centrality measures (DC, EC, SC) show high correlations whether in the original PIN or in the ensemble framework

### Prediction of low-degree essential proteins

Additional file 2: Tables S5-S8 present the top 100 proteins ranked by five centrality measures and by five ensemble methods on PIN24K and PIN76K. The degree distribution characteristics of these proteins are presented in Table 3. The average degree of the top 100 proteins ranked by each ensemble method is much smaller than that of its corresponding centrality measure, which is true on two yeast PINs. For example, the average degree of the top 100 proteins ranked by DC is 97.11, but it’s only 25.44 for those ranked by EnDC-a on PIN24K. On PIN76K, the average degrees for the top 100 proteins ranked by DC and EnDC-a are 327.57 and 94.21, respectively. This holds true for the other four centrality measures and their corresponding ensemble methods.

**Table 3:** Degree distribution characteristics among the top 100 proteins ranked by different centrality measures and different ensemble methods

		BC	EnBC-a	CC	EnCC-a	DC	EnDC-a	EC	EnEC-a	SC	EnSC-a
PIN76K	Average degree	306.32	135.99	277.71	108.88	327.57	94.21	290.7	93.37	326.98	94.54
	Minimal degree	17	10	24	6	138	20	83	23	127	20
	#low_degree	-	1	-	7	-	89	-	60	-	83
	#EP	-	0	-	2	-	75	-	52	-	69
	Average node strength	0.07	0.42	0.08	0.47	0.12	0.57	0.2	0.58	0.2	0.54
PIN24K	Average degree	93.23	37.63	89.96	26.51	97.11	25.44	79.14	21.43	97.04	24.82
	Minimal degree	33	4	33	4	64	4	31	3	60	4
	#low_degree	-	62	-	76	-	95	-	78	-	93
	#EP	-	47	-	64	-	78	-	64	-	78
	Average node strength	0.07	0.35	0.06	0.45	0.07	0.5	0.05	0.53	0.05	0.39

#low_degree denotes the number of proteins out of the top 100 ranked proteins by an ensemble method whose degrees are less than the minimal degree of the top 100 proteins ranked by the corresponding centrality measure, and #EP the number of essential proteins out of the #low_degree proteins

As shown in Table 3, more low-degree proteins are identified as essential candidates by the ensemble methods as compared with the corresponding centrality measures. For example, in the top 100 ranked proteins of EnDC-a, the degrees of 89 proteins are less than the minimal degree of the top 100 ranked proteins of DC on PIN76K, while 95 are low-degree proteins on PIN24K. Among the low-degree proteins identified by all five ensemble methods on PIN24K, about 75 % ~ 84 % are essential, and about 83 % ~ 86 % proteins are essential among the low-degree proteins identified by three ensemble methods (EnDC-a, EnEC-a and EnSC-a) on PIN76K. Few low-degree proteins are identified by EnBC-a and EnCC-a on PIN76K, compared with BC and CC, although the average degrees of the top 100 ranked proteins by EnBC-a and EnCC-a are much smaller than those of proteins ranked by BC and CC.

According to the definition of low-degree proteins, there is no intersection among the low-degree proteins and those ranked proteins by the corresponding centrality measures. Therefore, results in Table 3 also tell us that on PIN24K the overlap rates are very low between ensemble methods and their corresponding centrality measures, while this is true only for three pairs of methods (DC and EnDC-a, EC and EnEC-a, SC and EnSC-a) on PIN76K. Our further analysis on the proteins shown in Additional file 2: Tables S5-S8 indicate that the overlap rate between an ensemble method and its corresponding centrality measure is very low on two yeast PINs. The overlap rates are 2 % ~ 13 % on PIN24K and 5 % ~ 15 % on PIN76K. This low overlap indicates that the ensemble methods are quite different from their corresponding centrality measures.

In Table 3, we also included average node strength for each method, which was calculated based on the PCC-weighted PIN. In general, ensemble methods have much larger average node strengths than their corresponding centrality measures. This is consistent with Additional file 1: Figure S1, since essential proteins tend to have larger node strengths compared with nonessential proteins.

### Analysis on the different predicted proteins

**Fig. 5:**
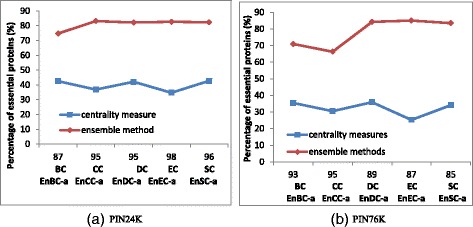
Comparison of the percentage of essential proteins out of the different predicted proteins between ensemble methods and their corresponding centrality measures. X-axis represents the number of different proteins in the top 100 proteins ranked by ensemble methods and their corresponding centrality measures, as well as the names for the ensemble methods and centrality measures. Y-axis represents the percentage of essential proteins out of the different predicted proteins

Since the overlap rate is very low between an ensemble method and its corresponding centrality measure, we evaluated the different proteins identified by each ensemble method and those by the corresponding centrality measure. The top 100 ranked proteins by different methods shown in Additional file 2: Tables S5-S8 are used for evaluation. Figure 5 shows how many essential proteins are predicted out of the different proteins identified by ensemble methods and those identified by the corresponding centrality measures. As expected, the results shown in Fig. 5 illustrate that the percentage of essential proteins identified by any ensemble method is consistently higher than that identified by the corresponding centrality measure. Take EnEC-a/EC as an example. Out of the top 100 ranked proteins identified by EnEC-a and by EC on PIN24K, 98 proteins are different. Out of the 98 proteins uniquely identified by EnEC-a, about 82.7 % proteins are essential. In contrast, only 34.7 % out of the 98 proteins uniquely identified by EC are essential. On PIN76K, for their top 100 ranked proteins, the numbers become 87, 85.1 % and 25.3 %, respectively. Similar results can be obtained for the remaining ensemble methods and their corresponding centrality measures on two yeast PINs.

Among the top 100 ranked proteins by different methods, there are several proteins which are not contained in standard-1122, but are contained in either or both of other two essential protein collections. For example, on PIN24K, two proteins (YBR106W and YBR200W) and three proteins (YCR088W, YFL023W and YBR106W) among the top 100 ranked proteins by BC and by EnBC-a respectively are not contained in standard-1122 but are contained in essential protein collection of SGD. YFL023W is a low-degree protein since its degree (equal to 10) is less than the minimal degree (equal to 33) of the top 100 proteins ranked by BC, but its node strength is high (3.17) which means that it is highly co-expressed with some of its interacting proteins (for example, YBR247C (0.93), YDL060W (0.95), YNL207W (0.94), YNL277W (0.65), the value is Pearson correlation). It’s interesting that all the four highly co-expressed partners are also essential proteins according to standard-1122. On PIN76K, there are more such proteins among the top 100 ranked proteins by five centrality measures than by the corresponding ensemble methods. For example, there are 9, 5, 6, 2, and 6 such proteins among the top 100 ranked proteins by BC, CC, DC, EC, and SC respectively, while there is only one such protein (YGR159C) among the top 100 ranked proteins by EnBC-a and zero for other four ensemble methods.

## Conclusions

The discovery of essential proteins at the network level is an important research field. Many network topology-based centrality measures for the discovery of essential proteins have been proposed. However, most of them are based on the topological properties of PINs and only have limited prediction accuracy. One reason might be the incomplete and noisy PINs on which the centrality measures depend. The other reason might be the fact that proteins’ essentiality are expected to relate to multiple biological features rather than one or few features captured by an individual centrality measure. Effective integration of multiple centrality measures as well as multiple biological data sources would be very useful for the task of identifying essential proteins. However, our experimental results for five commonly used centrality measures (DC, BC, EC, SC, and CC) on two yeast PINs (PIN76K and PIN24K) for *Saccharomyces cerevisiae* indicate that the overlap rate among the top ranked proteins by these centrality measures is very low, in addition to the low accuracy of these centrality measures.

To tackle the above problem, we propose an ensemble framework for the discovery of essential proteins, which is based on two ideas that essential proteins tend to be co-expressed with some of their interacting neighbors and that PPIs between two highly co-expressed proteins are expected to be more important to the proteins’ essentiality. Five commonly used centrality measures (BC, CC, DC, EC and SC) are used as base models. The performance of the ensemble framework has been evaluated on two yeast PINs (PIN24K and PIN76K). Experimental results indicate that the ensemble framework can greatly improve the prediction ability of five commonly used centrality measures, especially for top *n* ranked proteins with smaller *n* (i.e., n < 600). Furthermore, the overlap rate of essential proteins increases notably among the top proteins ranked by different ensemble methods. Essential proteins with low degree but highly co-expressed with some of their neighbors are more easily identified by ensemble methods than by centrality measures. It’s also interesting to note that the higher overlap rate of essential proteins between different ensemble methods does not translate to increased correlation but rather decreased correlation compared with the centrality measures. Therefore, the ensemble framework is valuable for predicting essential proteins from PINs. For organisms which have no available protein interaction data, computational methods for the construction of PINs [45] could give rise to a purely *in silico* network topology for predicting essential proteins.

The proposed ensemble framework can considerably improve the prediction accuracy of five centrality measures as well as the overlap rate between them for predicting essential proteins. However, the prediction accuracy decreases with the increase of *n*. The reason may be the fact that although essential proteins tend to be co-expressed with their interacting proteins, there are some essential proteins that have low co-expression levels with their interacting proteins. With the improvement in accuracy and completeness for both protein interaction data and gene expression data, we envisage that the effectiveness of the proposed ensemble framework might be enhanced accordingly.

In future research, more sophisticated ensemble frameworks will be explored for the discovery of essential proteins, especially the ensemble framework to integrate the predictive power from different topological properties as well as different types of biological information. Feature selection is often of a very important role in classification models. As reported in [46], features used to train the classifier determine its speed and performance. For identifying essential proteins across related organisms, especially distantly related organisms, feature selection should be more important since both the correlation level and predictive power of the features may vary in different organisms [35]. In the future, we will be also interested in developing feature selection method for identifying essential proteins from distantly related organisms.

## Abbreviations

BC, betweenness centrality; BC-thr, PCC-threshold method using BC as the base model; CC, closeness centrality; CC-thr, PCC-threshold method using CC as the base model; DC, degree centrality; DC-thr, PCC-threshold method using DC as the base model; EC, eigenvector centrality; EC-thr, PCC-threshold method using EC as the base model; EnBC-a, the ensemble method using absolute thresholding strategy and BC as the base model; EnBC-u, the ensemble method using uniform thresholding strategy and BC as the base model; EnCC-a, the ensemble method using absolute thresholding strategy and CC as the base model; EnCC-u, the ensemble method using uniform thresholding strategy and CC as the base model; EnDC-a, the ensemble method using absolute thresholding strategy and DC as the base model; EnDC-u, the ensemble method using uniform thresholding strategy and DC as the base model; EnEC-a, the ensemble method using absolute thresholding strategy and EC as the base model; EnEC-u, the ensemble method using uniform thresholding strategy and EC as the base model; EnSC-a, the ensemble method using absolute thresholding strategy and SC as the base model; EnSC-u, the ensemble method using uniform thresholding strategy and SC as the base model; maxPCC, maximal PCC; minPCC, minimal PCC; PCC, Pearson correlation coefficient; PIN24K, protein-protein interaction network from DIP database; PIN76K, protein-protein interaction network from BioGRID database; PINs, protein-protein interaction networks; PPI, protein-protein interaction; PPIs, protein-protein interactions; SC, subgraph centrality; SC-thr, PCC-threshold method using SC as the base model; wBC, PCC-weighted method using BC as the base model; wCC, PCC-weighted method using CC as the base model; wDC, PCC-weighted method using DC as the base model; wEC, PCC-weighted method using EC as the base model; wSC, PCC-weighted method using SC as the base model

## Additional files

Additional file 1:
**Figure S1.** The distributions of node strength for essential and nonessential protein. **Figure S2.** The distributions of co-expression weights for IBEPs and Non-IBEPs. **Figure S3.** Performance comparison of five centrality measures (BC, CC, DC, EC, and SC) on two yeast PINs (PIN24K and PIN76K) using uniform thresholding strategy ((a)-(h)). **Figure S4.** Relationship between the number of nonzero-degree nodes (or proteins) in PINs and the thresholds for generating the corresponding PINs using absolute thresholding strategy ((a)-(b)). **Figure S5.** Relationship between the number of nonzero-degree nodes (or proteins) in PINs and the thresholds for generating the PINs using uniform thresholding strategy ((a)-(b)). **Figure S6.** Performance comparison of five centrality measures (BC, CC, DC, EC, and SC) with their corresponding ensemble methods (absolute thresholding strategy) with different sample sizes or weights on two yeast PINs. **Figure S7.** Comparison of the number of essential proteins detected by each ensemble method using uniform thresholding strategy with different voting weights on two yeast PINs. **Table S1.** The number of common predicted proteins (overlap) among the top 100 proteins ranked by PCC-weighted methods. **Table S2.** The number of common predicted proteins (overlap) among the top 100 proteins ranked by single PCC-threshold methods (thr = 0.75). **Table S3.** Correlation between centrality measures based on their top 100 ranked proteins. **Table S4.** Correlation between ensemble methods based on their top 100 ranked proteins. (PDF 1294 kb) Additional file 2:
**Table S5-S6.** The information of the top 100 proteins ranked by five centrality measures and by five ensemble methods on PIN24K. **Table S7-S8.** The information of the top 100 proteins ranked by five centrality measures and by five ensemble methods on PIN76K. (XLSX 64 kb)

## References

[1] Winzeler EA, Shoemaker DD, Astromoff A, Liang H, Anderson K (1999). Functional characterization of the S. cerevisiae genome by gene deletion and parallel analysis. Science.

[2] Kamath RS, Fraser AG, Dong Y, Poulin G, Durbin R (2003). Systematic functional analysis of the Caenorhabditis elegans genome using RNAi. Nature.

[3] Steinmetz LM, Scharfe C, Deutschbauer AM, Mokranjac D, Herman ZS (2002). Systematic screen for human disease genes in yeast. Nature Gene.

[4] Dickerson JE, Zhu A, Robertson DL, Hentges KE (2011). Defining the role of essential genes in human disease. PLoS One.

[5] Hu W, Sillaots S, Lemieux S, Davison J, Kauffman S (2007). Essential gene identification and drug target prioritization in Aspergillus fumigatus. PLoS Pathog.

[6] Abadio AKR, Kioshima ES, Teixeira MM, Martins NF, Maigret B, Felipe MS (2011). Comparative genomics allowed the identification of drug targets against human fungal pathogens. BMC Genomics.

[7] Giaever G, Chu AM, Ni L (2002). Functional profiling of the Saccharomyces cerevisiae genome. Nature.

[8] Cullen LM, Arndt GM (2005). Genome-wide screening for gene function using RNAi in mammalian cells. Immunol Cell Biol.

[9] Roemer T, Jiang B, Davison J (2003). Large-scale essential gene identification in Candida albicans and applications to antifungal drug discovery. Mol Microbiol.

[10] Yu H, Greenbaum D, Lu HX, Zhu X, Gerstein M (2004). Genomic analysis of essentiality within protein networks. Trends Genet.

[11] Hahn MW, Kern AD (2004). Comparative genomics of centrality and essentiality in three eukaryotic protein interaction networks. Mol Biol Evol.

[12] Jeong H, Mason SP (2001). Lethality and centrality in protein networks. Nature.

[13] Yu H, Braun P, Yildirim MA (2008). High-quality binary protein interaction map of the yeast interactome network. Science.

[14] He X, Zhang J (2006). Why do hubs tend to be essential in protein networks?. PLoS Genet.

[15] Zotenko E, Mestre J, O’Leary DP, Przytycka TM (2008). Why do hubs in the yeast protein interaction network tend to be essential: reexamining the connection between the network topology and essentiality. PLoS Comput Biol.

[16] Ning K, Ng HK, Srihari S (2010). Examination of the relationship between essential genes in PPI network and hub proteins in reverse nearest-neighbor topology. BMC Bioinformatics.

[17] Vallabhajosyula R, Chakravarti D, Lutfeali S, Ray A, Raval A (2009). Identifying hubs in protein interaction networks. PLoS One.

[18] Joy M (2005). High-betweenness proteins in the yeast protein interaction network. J Biomed Biotechnol.

[19] Wuchty S, Stadler PF (2003). Centers of complex networks. J Theor Biol.

[20] Bonacich P (1987). Power and centrality: A family of measures. Am J Sociol.

[21] Estrada E, Rodríuez-Veláquez JA (2005). Subgraph centrality in complex networks. Phys Rev E.

[22] Li M, Zhang H, Wang J, Pan Y (2012). A new essential protein discovery method based on the integration of protein-protein interaction and gene expression data. BMC Syst Biol.

[23] Zhang X, Xu J, Xiao WX (2013). A new method for the discovery of essential proteins. PLoS One.

[24] Li M, Lu Y, Wang JX, Wu FX, Pan Y (2015). A topology potential-based method for identifying essential proteins from PPI networks. IEEE/ACM Trans. Comput. Biol. Bioinform..

[25] Li M, Wang JX, Chen X, Wang H, Pan Y (2011). A local average connectivity-based method for identifying essential proteins from the network level. Comput Biol Chem.

[26] Tang Y, Li M, Wang JX, Pan Y, Wu FX (2015). CytoNCA: a cytoscape plugin for centrality analysis and evaluation of biological networks. BioSystems.

[27] Acencio ML, Lemke N (2009). Towards the prediction of essential genes by integration of network topology, cellular localization and biological process information. BMC Bioinformatics.

[28] Li M, Wang J, Wang H, Pan Y (2013). Identification of essential proteins from weighted protein interaction networks. J Bioinform Comput Biol.

[29] Li M, Zheng R, Zhang H, Wang J, Pan Y: Effective Identification of essential proteins based on priori knowledge, network topology and gene expressions. Methods. doi:10.1016/j.ymeth.2014.02.016. 10.1016/j.ymeth.2014.02.016 24565748

[30] Wang J, Li M, Wang H, Pan Y (2012). Identification of essential proteins based on edge clustering coefficient. IEEE/ACM Trans. Comput. Biol. Bioinform..

[31] Ren J, Wang JX, Li M, Wu FX (2015). Discovering essential proteins based on PPI network and protein complex. Int J Data Min Bioinform.

[32] Li M, Lu Y, Niu ZB, Wu FX: United complex centrality for identification of essential proteins from PPI networks. IEEE/ACM Trans Comput Biol Bioinform. doi:10.1109/TCBB.2015.2394487. 10.1109/TCBB.2015.2394487 28368815

[33] Zhao BH, Wang JX, Li M, Wu FX, Pan Y (2014). Prediction of essential proteins based on overlapping essential modules. IEEE Trans. Nanobioscience.

[34] Wang JX, Peng W, Wu F (2013). Computational approaches to predicting essential proteins: A survey. Proteomics Clin.

[35] Zhang X, Acencio ML, Lemke N: Predicting essential genes and proteins based on machine learning and network topological features: a comprehensive review. Front. Physiol. doi:10.3389/fphys.2016.00075. 10.3389/fphys.2016.00075 PMC4781880 27014079

[36] Xiao Q, Wang J, Peng X, Wu FX, Pan Y: Identifying essential proteins from active PPI networks constructed with dynamic gene expression. BMC Genomics. 2015; 16 Suppl 3:S1. Epub 2015/02/25. doi:10.1186/1471-2164-16-S3-S1 PMID: 25707432; PubMed Central PMCID: PMC4331804. 10.1186/1471-2164-16-S3-S1 PMC4331804 25707432

[37] Ernesto E. Virtual identification of essential proteins within the protein interaction network of yeast. Proteomics. 2006;6(1):35–40. 10.1002/pmic.200500209 16281187

[38] Xenarios I, Rice DW, Salwinski L, Baron MK, Marcotte EM, Eisenberg D (2000). DIP: the database of interacting proteins. Nucleic Acids Res.

[39] Stark C, Breitkreutz BJ, Reguly T, Boucher L, Breitkreutz A, Tyers M. BioGRID: A General Repository for Interaction Datasets. Nucleic Acids Res. 2006;34:D535–9 10.1093/nar/gkj109 PMC1347471 16381927

[40] Zhang R, Lin Y (2009). DEG 5.0, a database of essential genes in both prokaryotes and eukaryotes. Nucleic Acids Res.

[41] Cherry JM (1988). SGD: Saccharomyces Genome Database. Nucleic Acids Res.

[42] Tu BP, Kudlicki A, Rowicka M, McKnight SL (2005). Logic of the yeast metabolic cycle: temporal compartmentalization of cellular processes. Science.

[43] Rokach L (2010). Ensemble-based classifiers. Artif Intell Rev.

[44] Wittmeyer J, Joss L, Formosa T (1999). Spt16 and Pob3 of Saccharomyces cerevisiae form an essential, abundant heterodimer that is nuclear, chromatin-associated, and co-purifies with DNA polymerase alpha. Biochemistry.

[45] Singh R, Park D, Xu J, Hosur R, Berger B: Struct2Net: a web service to predict protein-protein interactions using a structure-based approach. Nucleic Acids Research, 2010, 38. doi:10.1093/nar/gkq481. 10.1093/nar/gkq481 PMC2896152 20513650

[46] Zhong JC, Wang JX, Peng W, Zhang Z, Li M (2015). A feature selection method for prediction essential protein. Tsinghua Sci Technol.

