# Supplementary material for: An ensemble framework for identifying essential proteins
Source: BMC Bioinformatics. 2016 Aug 25;17(1):322. doi: 10.1186/s12859-016-1166-7 (PMC4997703; doi:10.1186/s12859-016-1166-7)
Supplement: Additional file 1: — Figure S1. The distributions of node strength for essential and nonessential protein. Figure S2. The distributions of co-expression weights for IBEPs and Non-IBEPs. Figure S3. Performance comparison of five centrality measures (BC, CC, DC, EC, and SC) on two yeast PINs (PIN24K and PIN76K) using uniform thresholding strategy ((a)-(h)). Figure S4. Relationship between the number of nonzero-degree nodes (or proteins) in PINs and the thresholds for generating the corresponding PINs using absolute thresholding strategy ((a)-(b)). Figure S5. Relationship between the number of nonzero-degree nodes (or proteins) in PINs and the thresholds for generating the PINs using uniform thresholding strategy ((a)-(b)). Figure S6. Performance comparison of five centrality measures (BC, CC, DC, EC, and SC) with their corresponding ensemble methods (absolute thresholding strategy) with different sample sizes or weights on two yeast PINs. Figure S7. Comparison of the number of essential proteins detected by each ensemble method using uniform thresholding strategy with different voting weights on two yeast PINs. Table S1. The number of common predicted proteins (overlap) among the top 100 proteins ranked by PCC-weighted methods. Table S2. The number of common predicted proteins (overlap) among the top 100 proteins ranked by single PCC-threshold methods (thr = 0.75). Table S3. Correlation between centrality measures based on their top 100 ranked proteins. Table S4. Correlation between ensemble methods based on their top 100 ranked proteins. (PDF 1294 kb) [file 12859_2016_1166_MOESM1_ESM.pdf]

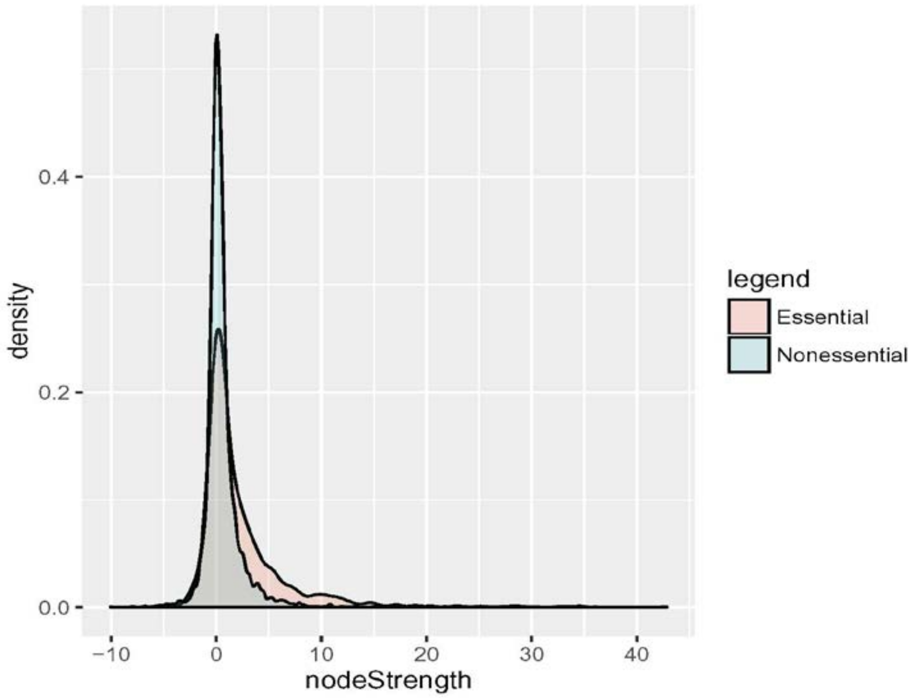

(a) PIN24K

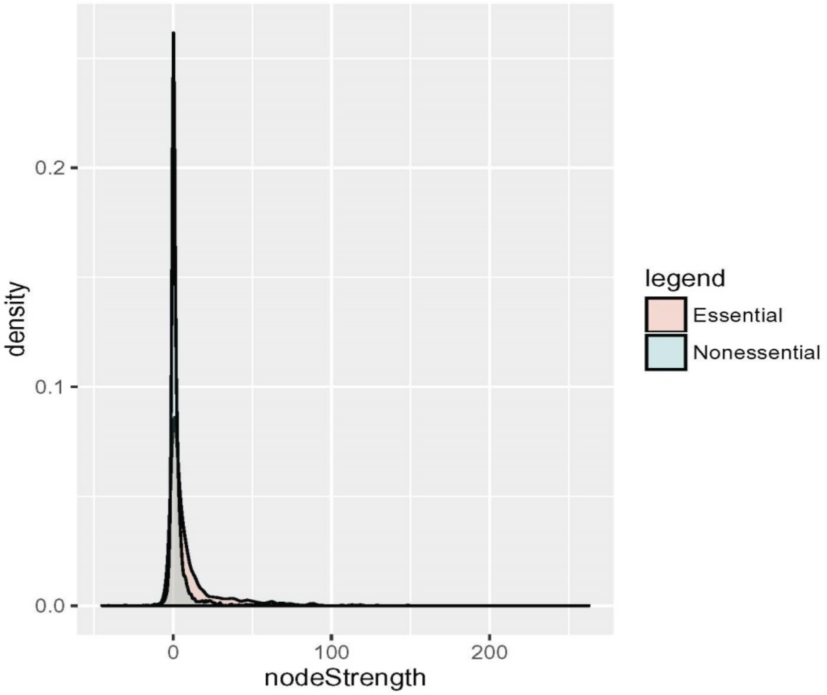

(b) PIN76K

Figure S1. The distributions of node strength for essential and nonessential proteins. Node strength of a protein here was calculated as the sum of the edge weights (co-expression weight) appending on it.

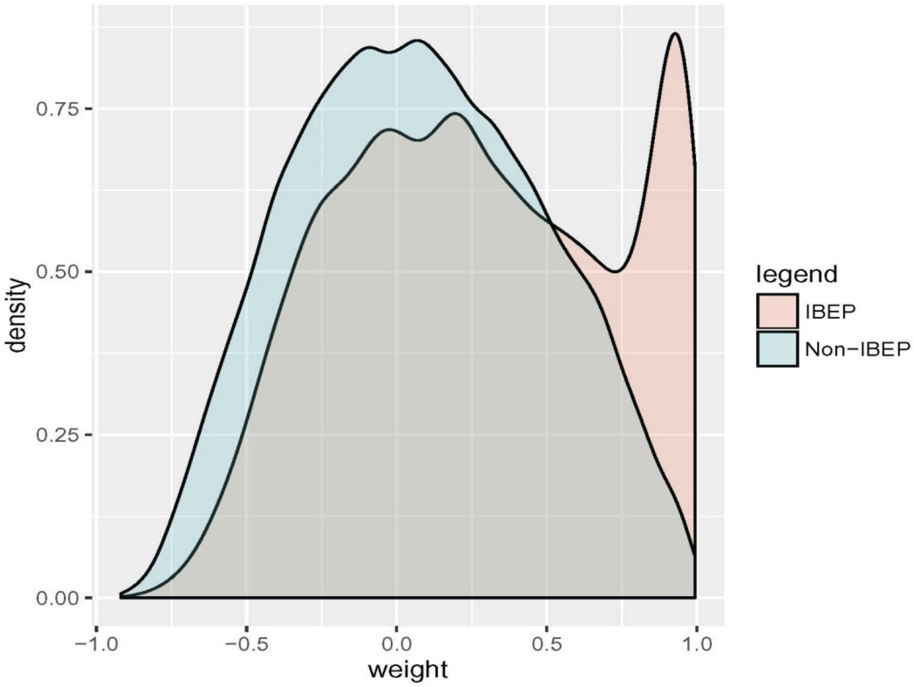

(a) PIN24K

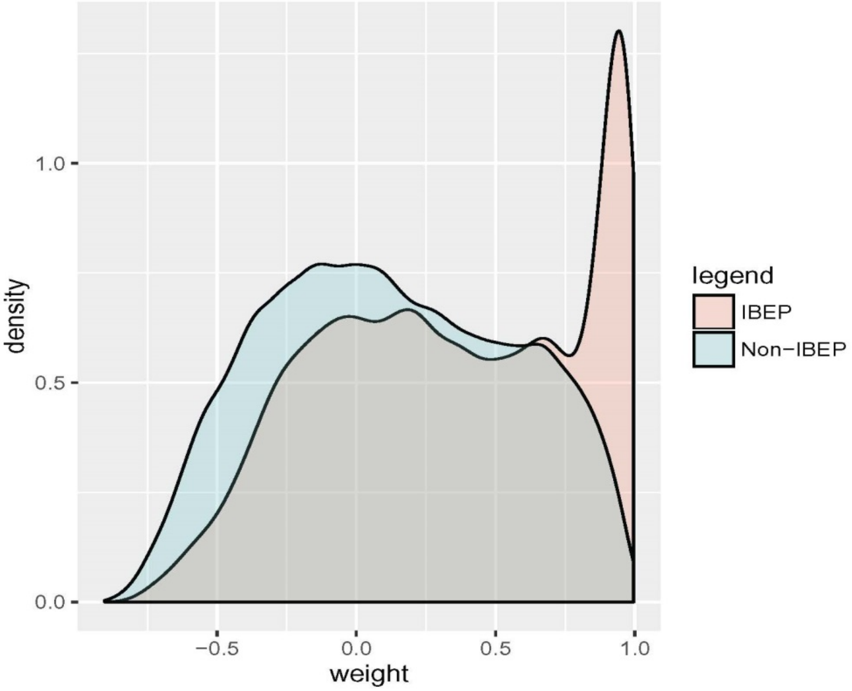

(b) PIN76K

Figure S2. The distributions of co-expression weights for IBEPs and Non-IBEPs. IBEP means interaction between two essential proteins. The co-expression weight for each proteins pair was calculated as the Pearson correlation coefficient.

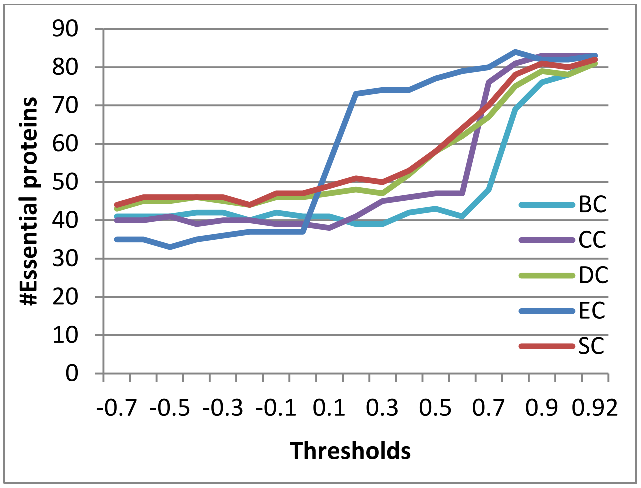

(a)Top 100 ranked proteins (PIN24K)

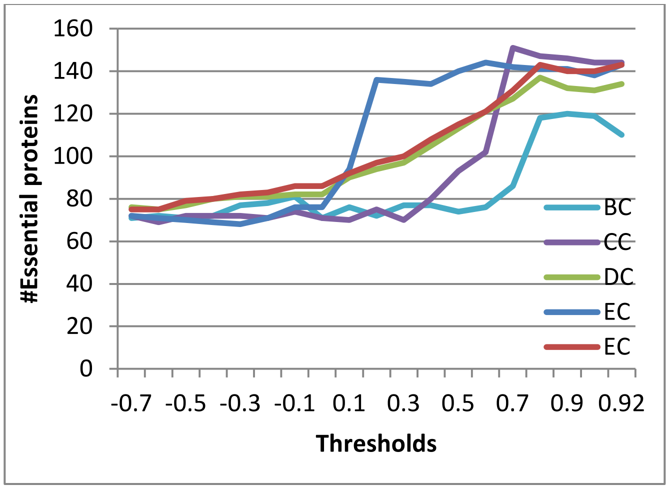

(b) Top 200 ranked proteins (PIN24K)

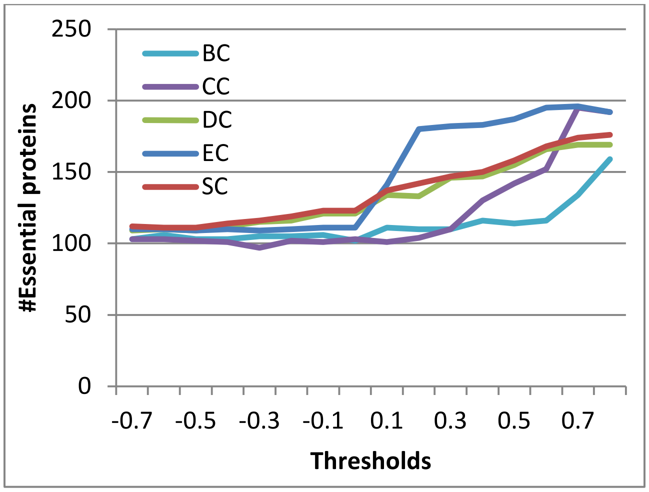

(c)Top 300 ranked proteins (PIN24K)

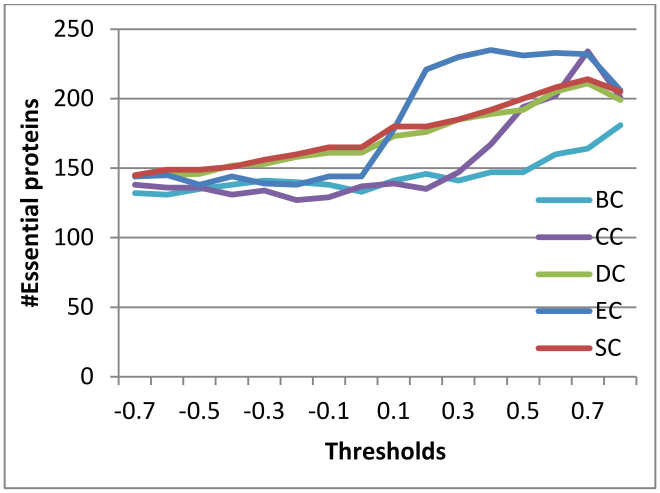

(d) Top 400 ranked proteins (PIN24K)

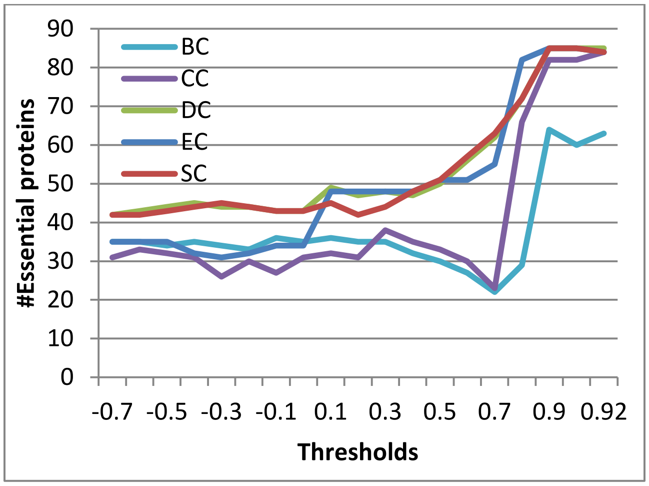

(e)Top 100 ranked proteins (PIN76K)

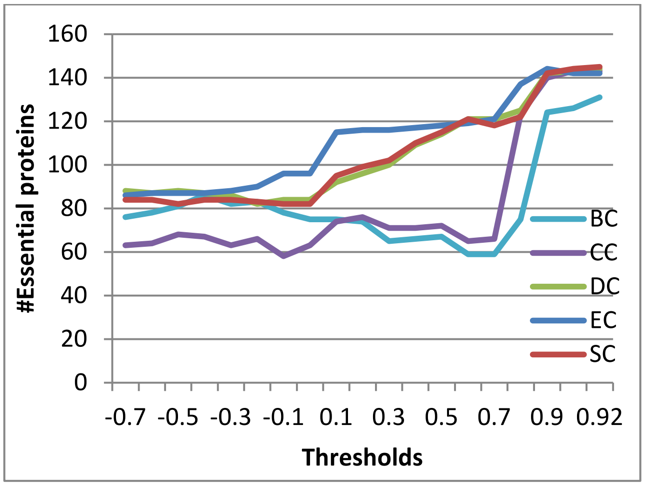

(f) Top 200 ranked proteins (PIN76K)

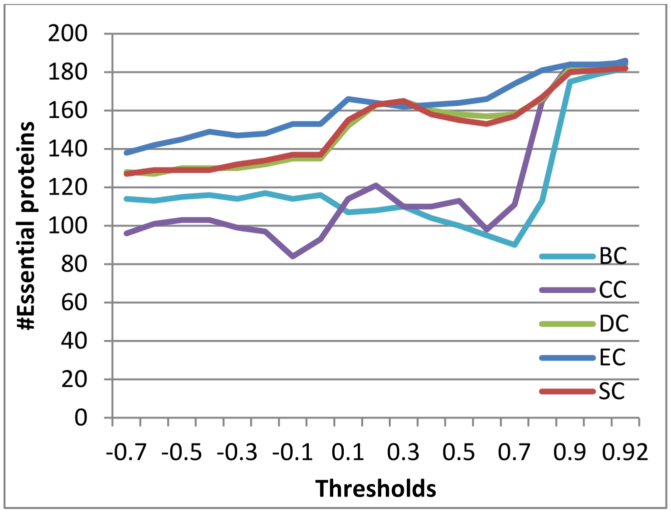

(g)Top 300 ranked proteins (PIN76K)

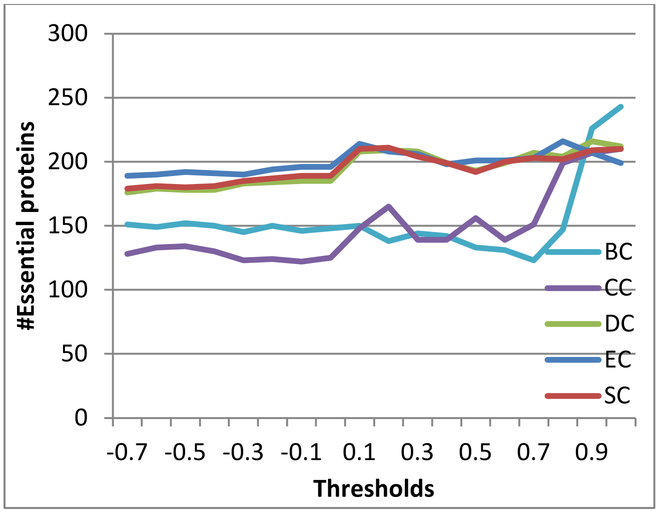

(h) Top 400 ranked proteins (PIN76K)

Figure S3. Performance comparison of five centrality measures (BC, CC, DC, EC, and SC) on two yeast PINs (PIN24K and PIN76K) using uniform thresholding strategy. For each yeast PIN (PIN24K or PIN76K), 19 PINs are generated by using the uniform thresholding strategy (see Methods). Proteins are ranked according to their values calculated by each centrality measure on each PIN. For each centrality measure on each PIN, top n proteins are selected as candidates for essential proteins, out of which the number of true essential proteins are determined. X-axis represents the thresholds and y-axis the number of true essential proteins in top n ranked proteins. Proteins contained in standard-1122 are considered as essential proteins.

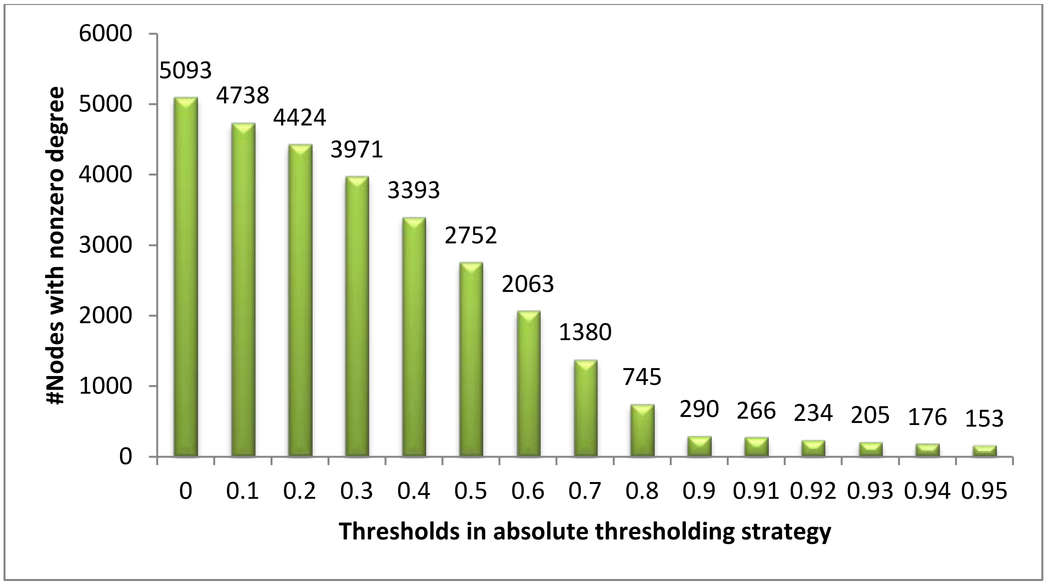

(a) PIN24K

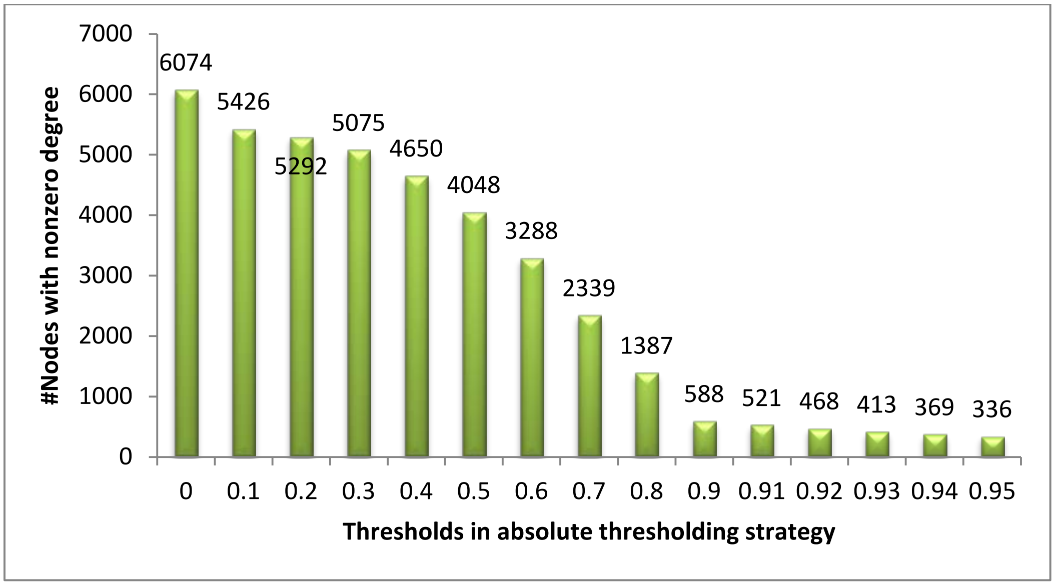

(b)PIN76K

Figure S4. Relationship between the number of nonzero-degree nodes (or proteins) in PINs and the thresholds for generating the corresponding PINs using absolute thresholding strategy. X-axis represents the thresholds in absolute thresholding strategy for generating the PINs and y-axis the number of nodes with nonzero degree in the corresponding PINs.

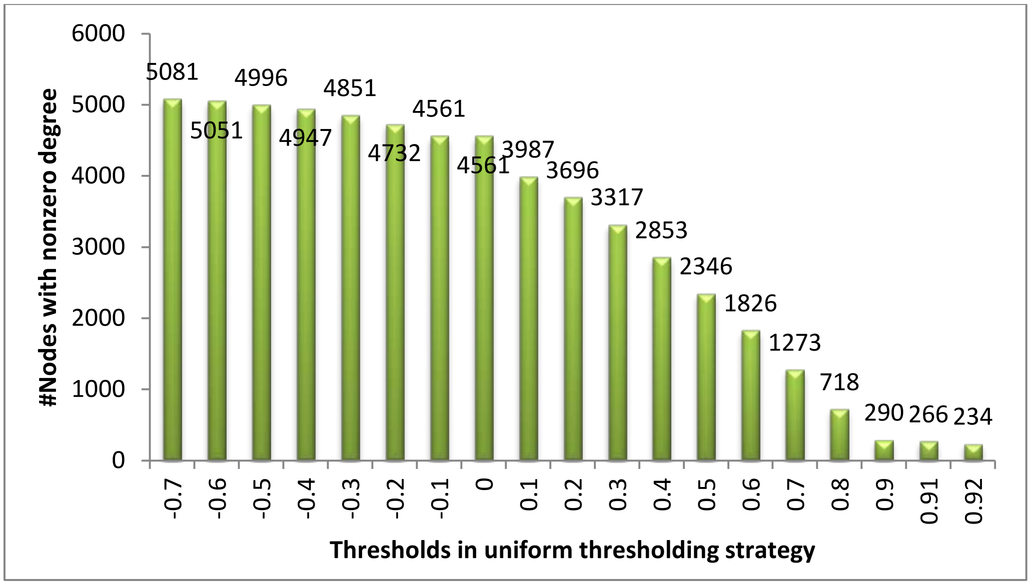

(a) PIN24K

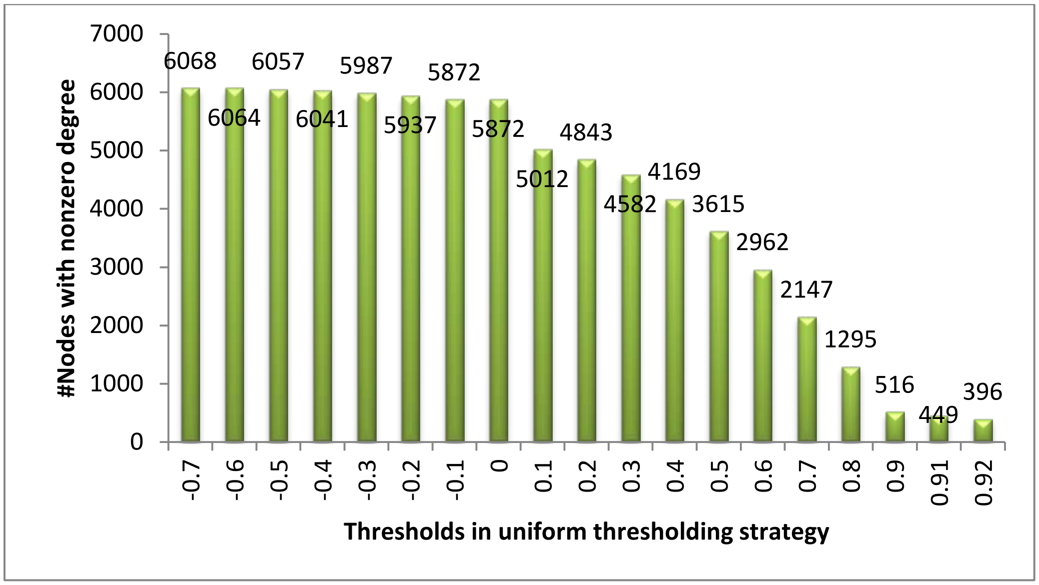

(b) PIN76K

Figure S5. Relationship between the number of nonzero-degree nodes (or proteins) in PINs and the thresholds for generating the PINs using uniform thresholding strategy. X-axis represents the thresholds in uniform thresholding strategy for generating the PINs and y-axis the number of nodes with nonzero degree in the corresponding PINs.

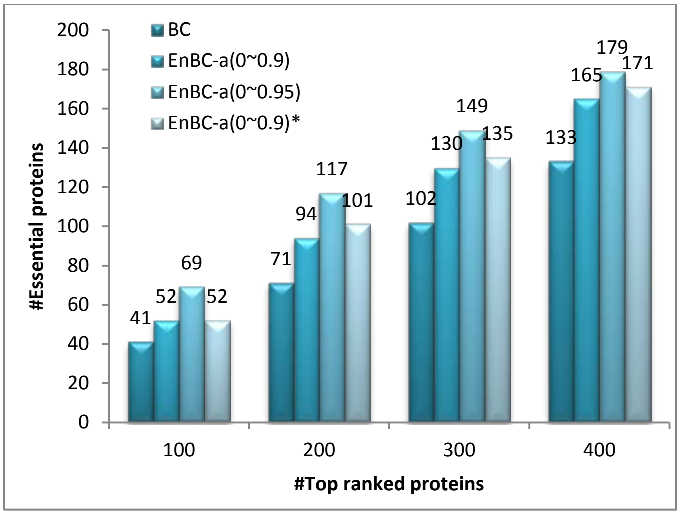

(a) BC and EnBC-a on PIN24K

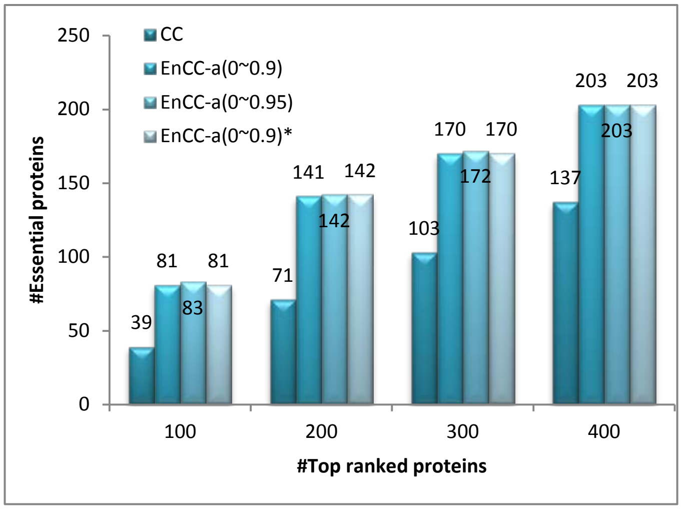

(b) CC and EnCC-a on PIN24K

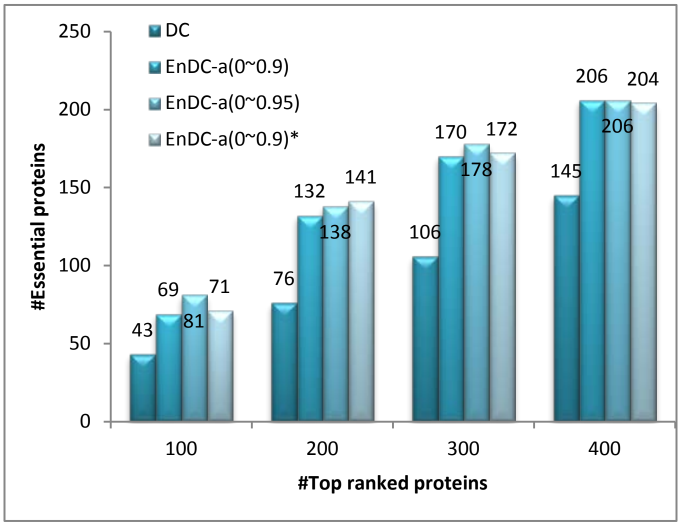

(c) DC and EnDC-a on PIN24K

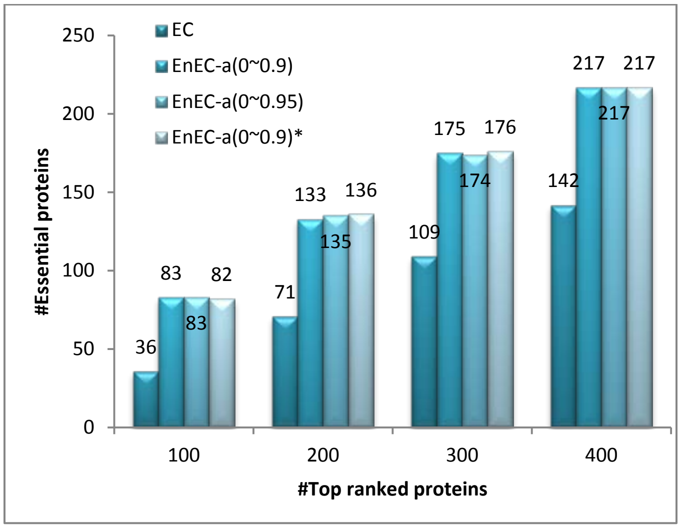

(d) EC and EnEC-a on PIN24K

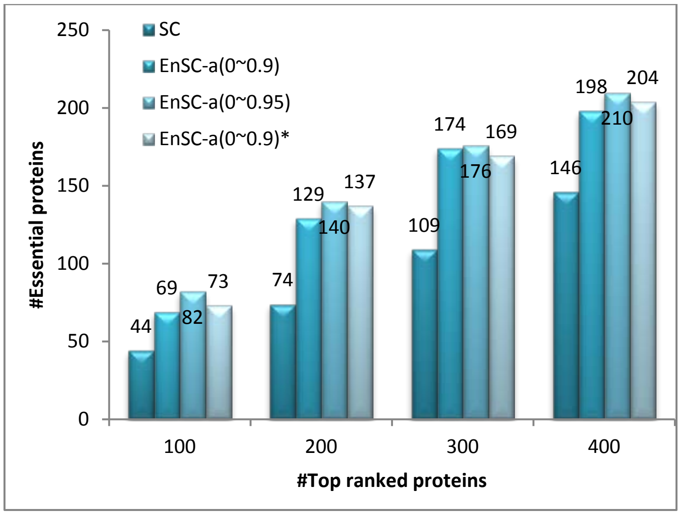

(e) SC and EnSC-a on PIN24K

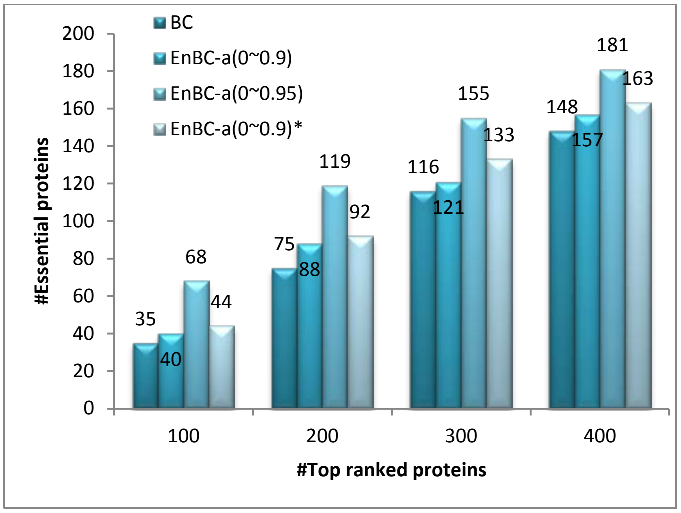

(f) BC and EnBC-a on PIN76K

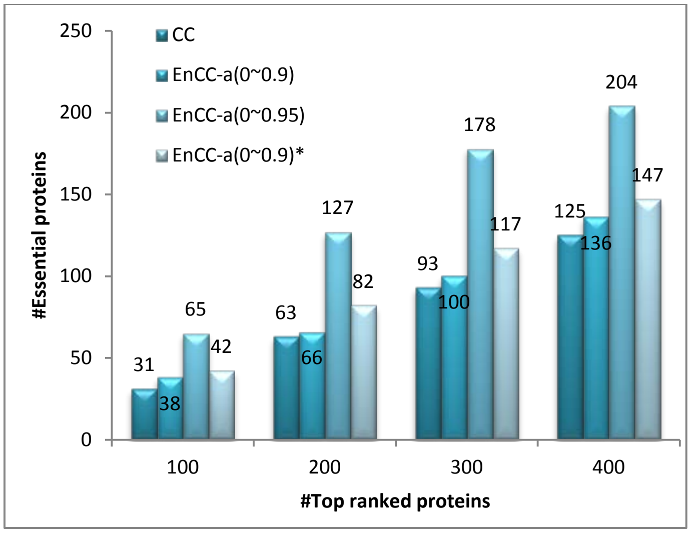

(g) CC and EnCC-a on PIN76K

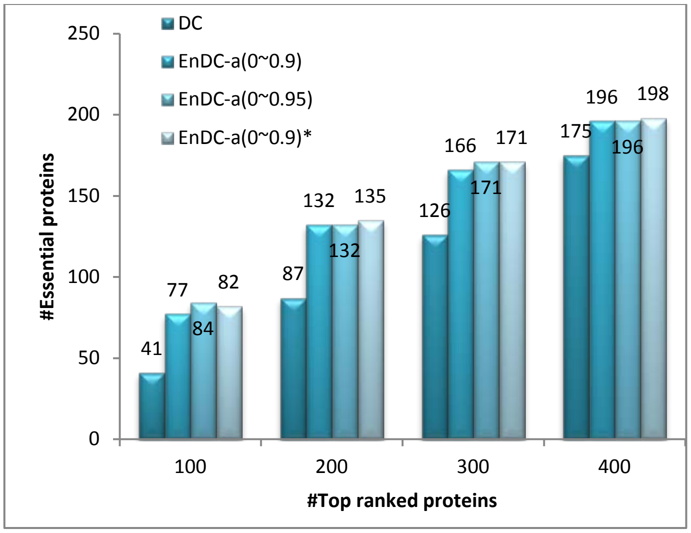

(h) DC and EnDC-a on PIN76K

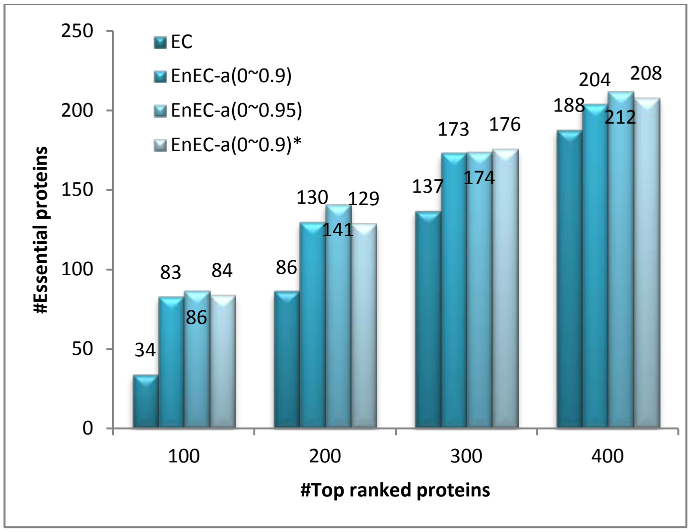

(i) EC and EnEC-a on PIN76K

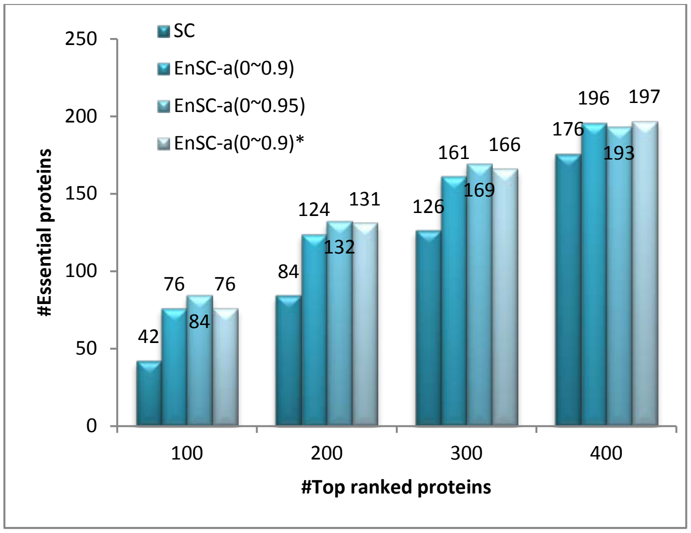

(j) SC and EnSC-a on PIN76K

Figure S6. Performance comparison of five centrality measures (BC, CC, DC, EC, and SC) with their corresponding ensemble methods (absolute thresholding strategy) with different sample sizes or weights on two yeast PINs (PIN24K and PIN76K). For each yeast PIN (PIN24K or PIN76K), 15 PINs are generated by using the absolute thresholding strategy (see Methods) with thresholds thr={0, 0.1, 0.2, …, 0.9, 0.91, 0.92, .., 0.95}. M(0~0.9) denotes the ensemble method only using the first 10 generated PINs (with thresholds from 0 to 0.9 and the corresponding weights w={1 2 3 5 8 13 21 34 55 89}), while M(0~0.95) the ensemble method using the 15 generated PINs with weights w={1 2 3 5 8 13 21 34 55 89 144 233 377 610 987}. M(0~0.9)* denotes the ensemble method only using the first 10 generated PINs with thresholds from 0 to 0.9 and the corresponding weights w={1 2 3 5 8 13 21 34 55 200}. M is one of the five ensemble methods: EnBC-a, EnCC-a, EnDC-a, EnEC-a, and EnSC-a. Proteins are ranked according to their scores calculated either by each centrality measure or by each ensemble method. For each method, top *n* ranked proteins are selected as candidates for essential proteins, out of which the number of true essential proteins are determined. X-axis represents the number of top *n* ranked proteins and y-axis the number of true essential proteins in top *n* ranked proteins. Proteins contained in standard-1122 are considered as essential proteins.

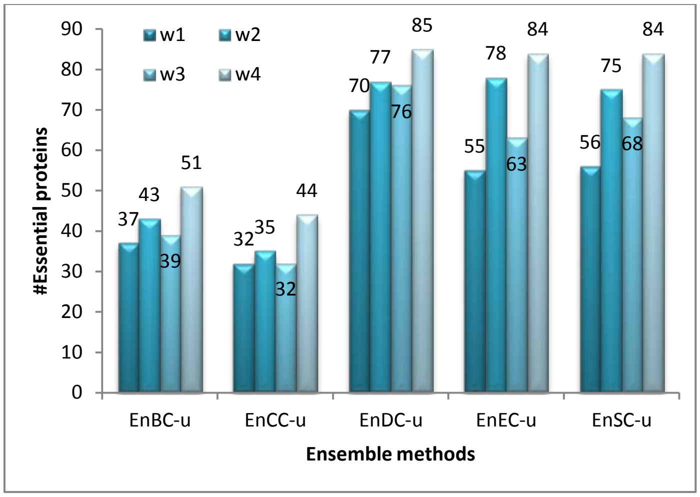

(a) Top 100 ranked proteins on PIN76K

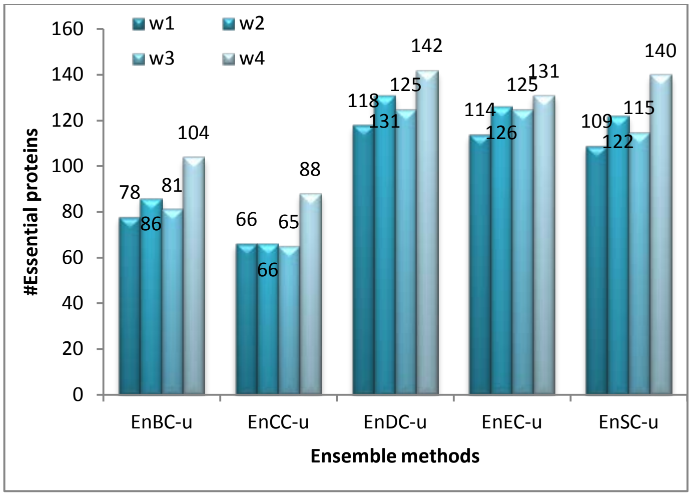

(b) Top 200 ranked proteins on PIN76K

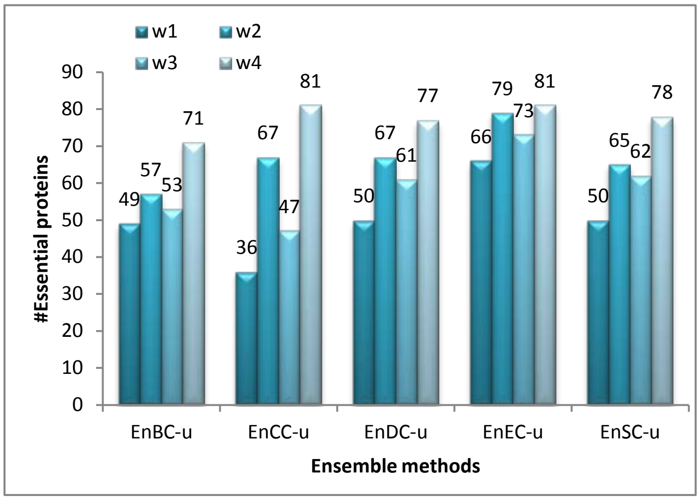

(c) Top 100 ranked proteins on PIN24K

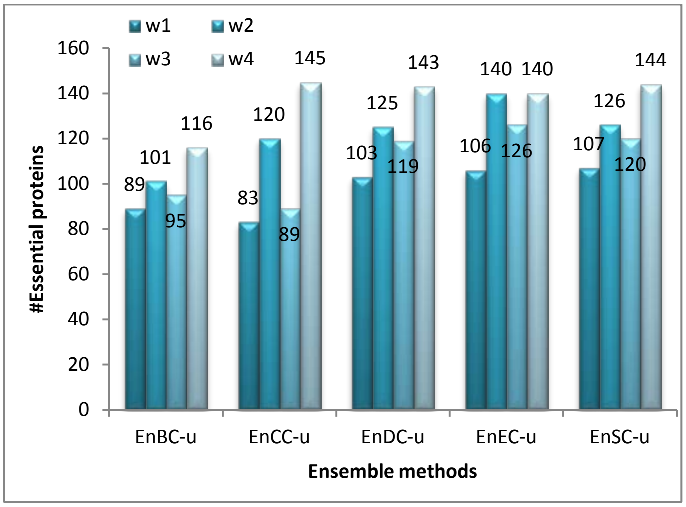

(d) Top 200 ranked proteins on PIN24K

Figure S7. Comparison of the number of essential proteins detected by each ensemble method using uniform thresholding strategy with different voting weights on two yeast PINs. w1={1 1 1 1 1 1 1 1 1 1 1 1 1 1 1 1 1 1 1}, is used as a base line since it doesn't differentiate each generated PINs. w2={ 1 1 1 1 1 1 1 1 8 9 10 11 12 13 15}, is called as prior guided advance weighting. w3={1 2 3 4 5 6 7 8 9 10 11 12 13 14 15 16 17 181 19}, is called as gradual advance weighting. w4={1 1 1 1 1 1 1 1 2 3 5 8 13 21 34 55 89 144 233}, is called as prior guided dominant weighting in which the latter generated PIN's weight is the sum of its adjacent two former PINs' weights for PINs generated with thresholds larger than zero and the weights for other PINs are set to 1.

Table S1. **The number of common predicted proteins (overlap) among the top 100 proteins ranked by PCC-weighted methods.** Proteins are ranked according to the scores calculated by each method. The top 100 ranked proteins for each method are selected as the candidates for essential proteins, out of which the number of true essential proteins are determined. #overlap represents the number of common predicted proteins among the top 100 ranked proteins by different methods and #EP the number of true essential proteins among the overlaps.

Datasets	PCC-weighted methods	wEC		wSC		#overlaps among three weighted methods	
		#overlap	#EP	#overlap	#EP	#overlap	#EP
PIN76K	wDC	84	47	65	30	61	27
	wEC			64	27		
PIN24K	wDC	53	40	15	11	4	3
	wEC			9	5		

Table S2. **The number of common predicted proteins (overlap) among the top 100 proteins ranked by single PCC-threshold methods (thr=0.75).** Proteins are ranked according to the scores calculated by each method. The top 100 ranked proteins for each method are selected as the candidates for essential proteins, out of which the number of true essential proteins are determined. #overlap represents the number of common predicted proteins among the top 100 ranked proteins by different methods and #EP the number of true essential proteins among the overlaps.

Datasets	PCC-threshold methods	BC-thr		EC-thr		SC-thr		CC-thr		#overlaps among five centrality measures	
		#overlap	#EP	#overlap	#EP	#overlap	#EP	#overlap	#EP	#overlap	#EP
PIN76K	DC-thr	20	10	83	62	83	62	39	18	13	7
	BC-thr			15	9	15	9	33	12		
	EC-thr					100	76	30	16		
	SC-thr							30	16		
PIN24K	DC-thr	43	24	58	48	58	48	56	42	16	14
	BC-thr			18	15	17	15	42	21		
	EC-thr					98	80	57	48		
	SC-thr							55	47		

Table S3. Correlation between centrality measures based on their top 100 ranked proteins. Pearson correlation was used.

	Centrality measures	CC	DC	EC	SC
PIN76K	BC	0.745	0.913	0.707	0.862
	CC		0.807	0.677	0.768
	DC			0.877	0.93
	EC				0.971
PIN24K	BC	0.352	0.842	-0.022	0.057
	CC		0.549	0.579	0.625
	DC			0.335	0.437
	EC				0.983

Table S4. Correlation between ensemble methods based on their top 100 ranked proteins. Pearson correlation was used.

	Ensemble methods	EnCC-a	EnDC-a	EnEC-a	EnSC-a
PIN76K	EnBC-a	0.055	0.12	0.022	0.094
	EnCC-a		0.042	-0.036	0.052
	EnDC-a			0.869	0.863
	EnEC-a				0.975
PIN24K	EnBC-a	0.173	0.651	0.356	0.409
	EnCC-a		0.094	0.047	0.13
	EnDC-a			0.789	0.793
	EnEC-a				0.943
